# APOLLO-Live: A multi-criteria-based webtool for synchronous group decision making and consensus reaching in energy and climate policy deliberations

**DOI:** 10.12688/openreseurope.19614.1

**Published:** 2025-03-31

**Authors:** Konstantinos Koasidis, Anastasios Soursos, Georgios Xexakis, Álvaro Labella, Anastasios Karamaneas, Alexandros Nikas

**Affiliations:** 1 Energy Policy Unit, School of Electrical and Computer Engineering, National Technical University of Athens, Zografou, Attica, 15780, Greece; 2 Forecasting & Strategy Unit, School of Electrical and Computer Engineering, National Technical University of Athens, Zografou, Attica, 15780, Greece; 3 HOLISTIC P.C., Athens, 15343, Greece; 4 Department of Computer Science, Universidad de Jaén, Jaén, Andalusia, 23071, Spain

**Keywords:** Group decision making, consensus reaching, multi-criteria decision aid, stakeholder engagement, deliberation, climate action, just transition

## Abstract

Pursuing a just, equitable, and desirable sustainable transition requires stakeholders from a diverse set of backgrounds and varying viewpoints to actively engage in deliberations to co-design their future. Facilitating stakeholder engagement activities has so far relied on either qualitative frameworks and processes or simplified quantitative approaches such as surveys, which render eliciting tangible and actionable outcomes challenging. Although group decision making and consensus reaching can leverage the capacity of multi-criteria decision aid to address this gap, tools implementing such processes remain scarce.

Here, we present APOLLO-Live, a stakeholder engagement webtool that can be used live in workshops to facilitate deliberations in energy and climate policy. The tool relies on linguistic variables, which are easily comprehensible by the participants, and employs the 2-tuple TOPSIS group decision making method to prioritise needs faced by different communities, as well as solutions that can be implemented to advance the energy transition. It also fleshes out differences in the voting patterns of different groups of voters and calculates a consensus metric to shed light on conflicts arising. Through tips and suggestions provided within the tool and based on multiple rounds of voting, supplemented by live discussions during the workshop, the tool can improve consensus and synthesise multiple perspectives in the produced outcomes, assisting towards bridging the conflicts and producing solutions that are widely accepted.

We highlight the functionalities of the webtool, including how it can be used to advance stakeholder dialogues, in two use cases targeting the preferences of the youth in terms of (a) actions to advance climate action and (b) investments to be prioritised. Finally, although the development and use principles followed focused on the energy and climate policy domain, the tool can be used in any application where multi-criteria decision aid and group decision making can potentially be employed.

## Plain Language Summary

Climate change is a serious threat to humanity. However, not all people agree on the best way to address it, since neither the impacts of climate change nor the potential benefits of the green transition are evenly and fairly distributed. As such, it is important that all people become an active part of the discussions and debates. Here, we present APOLLO-Live, an online tool that can be used live in workshops to assist such discussions. For example, it can be used to assist voters in highlighting the most important challenges they face in their communities, as well as indicate solutions, such as investments, that should be urgently implemented. The tool aims to improve the agreement between participants, based on multiple rounds of voting, discussions, and displayed suggestions. A core component of the tool lies in understanding how the opinions of different groups of people differ (e.g., citizens compared to academics or policymakers). The methodologies employed offer a seamless use of the tool by both the participants and potential organisers of workshops. We also present two use case examples focusing on understanding the preferences of young people in questions related to climate action, explaining how the tool can be used, and discussing what types of results can be produced. Despite our focus on themes related to climate change, the tool can be used in various domains and decision-making in the context of conflicting viewpoints.

## Introduction

Involving and interacting with stakeholders within the scientific process has been gaining traction ^1^, not to mention the increasingly acknowledged need to better communicate scientific findings to broader audiences as well as enable implementation of these findings in real-life applications ^2^. Although stakeholder theory has its roots in the business world ^3^, as a means for businesses and entities to engage with those affected by their decisions ^4^ and in the essence of corporate social responsibility ^5, 6^, stakeholder engagement has found applications across many fields that are relevant to sustainability ^7^ including environmental conservation ^8, 9^ and decarbonisation ^10^, and provided support to both scientific and policymaking processes ^11, 12^.

Especially with regard to climate change mitigation, and particularly upon raising aspects of justice and fairness, stakeholder engagement is of utmost importance: the envisaged energy transitions, albeit vital to ensure environmental conservation and addressing climate change, are expected to also have negative impacts on national and regional scales—for example, on communities that have hitherto thrived on fossil fuel development (extraction, use, export, etc.). Similarly, inequalities between and within countries may be exacerbated as a result of climate change impacts or distributional implications of climate policies ^13^. As such, all stakeholders—citizens included—affected the most must be part of the process that gives shape to their future; their inclusion can help towards societal acceptance and legitimise the required transformations ^14^.

More often than not, however, stakeholders are not a homogeneous group of people that share a common vision for their future, nor for the right pathway to that. This conflict of viewpoints, strategies, motives, and concerns can spark vivid debates over the desired course of action across multiple geographical scales (e.g., climate negotiations among countries, national policy consultations, municipal planning deliberations, etc.)—e.g., on the timeline of the transition, policies and required investments, eligible technologies, or the allocation of funds ^15, 16^. When deliberating such topics, and in particular when engaging with affected communities, it is therefore important to flesh out and highlight these different priorities and preferences, as well as attempt to bridge conflicting viewpoints and converge to widely acceptable paths through compromises.

Despite this increasingly acknowledged importance to include stakeholders in energy and climate policymaking as well as in scientific processes underpinning it, deliberation and co-creation activities with the aim to accommodate and synthesise different perspectives, and make progress towards bridging said conflicts remain scarce ^17^. Most existing efforts focus on producing stakeholder-informed outputs through deliberation exercises ^18, 19^ or asynchronous elicitation activities—for instance, with the use of multi-criteria decision aid (MCDA) approaches ^20– 22^. MCDA techniques, especially within the context of group decision making (GDM) and consensus measuring/reaching, are considered very useful in engaging with stakeholders due to their ability to incorporate multiple dimensions of any studied aspect, while maintaining comprehensibility in terms of their interaction with participants providing their feedback ^23^. Yet, despite numerous tools existing in the MCDA literature ^24– 30^, only a handful have been used synchronously in workshops ^31– 33^, in a way that resolves conflicts and approaches consensus live, and only in context-specific and thus non-generalisable applications.

To address this gap, this paper introduces APOLLO-Live, a GDM- and MCDA-based interactive webtool designed for stakeholder engagement during live workshops. The tool enables stakeholders to voice their opinions, assists in reconciling diverse and potentially conflicting perspectives and viewpoints, and facilitates prioritisation of needs and evaluation of solutions. Developed within the principles of deliberative democracy, the processes followed in the tool aim to enable equitable, fair, and inclusive transitions in collaboration with stakeholders.

## Methods

### Overview and theoretical concepts of the APOLLO-Live stakeholder engagement webtool

Drawing from an existing asynchronous MCDA tool ^20, 34^, APOLLO-Live constitutes a significant expansion in the field of MCDA tools for stakeholder engagement, primarily in that it can be used live in workshops to allow stakeholders to express their opinions, as well as to bridge conflicts in participatory workshops through consensus reaching. The following concepts are core in the development and use of the tool:

•   Alternatives: A set of options that the stakeholders vote on and evaluate.

•   Criteria: The dimensions against which each alternative is evaluated by the stakeholders. The selection of criteria should ensure that the principle of a “consistent family of criteria” ^35^ is respected, to ensure comprehensibility and that all necessary dimensions are accounted.

•   Vote: Each stakeholder provides their evaluations using non-negative Likert-type linguistic scales—e.g., {Very Low, Low, Medium, High, Very High}—which can be used to perform semi-quantitative calculations.

•   Ranking: Different MCDA techniques offer different outputs (i.e., selection, ranking, classification). Those typically employed in GDM, and as such in the APOLLO-Live webtool, comprise mostly of aggregation and/or distance-based approaches that offer a ranking of the alternatives, the translation of which depends on the selection of criteria.

•   Consensus: A metric expressing the agreement and proximity—in line with the distance-based focus of the MCDA framework—in the votes between the group of stakeholders. In this context, consensus refers to ‘soft consensus’ ^36, 37^, defined as the level of agreement, rather than a ‘hard consensus’—which is instead defined as a unanimous agreement that is almost impossible to achieve ^38^.

In particular, stakeholders participate by casting their votes on different options through a fit-for-purpose and completely customisable survey-style questionnaire. To increase the comprehensibility of the aforementioned concepts, we can consider the following working example: Stakeholder *S _k_* might consider (and vote accordingly) that the construction of a factory for producing components for renewable energy plants [Alternative *S _i_*] could potentially have a ‘High’ impact [Stakeholder’s vote following a linguistic scale] on regional development [Criterion *C _j_*]. The tool then aggregates all individual votes—namely the evaluation of the impact of all alternatives to all established criteria independently and from all stakeholders—and then generates a collective solution that reflects the group’s overall perspective. This outcome is essentially a ranking of alternatives, ordered from the most to the least significant/impactful, based on how they are evaluated by the participants across the criteria. Returning to the previous working example, if most stakeholders believe that the factory could indeed have an equally high impact not only on regional development but on other dimensions as well (e.g., job creation), this alternative is expected to rank among the top positions with a qualitative evaluation of high importance. The tool also identifies the level of (dis-) agreement between the participants and offers tailored tips to improve the consensus among the stakeholders in multiple rounds of voting.

**Figure 1.**
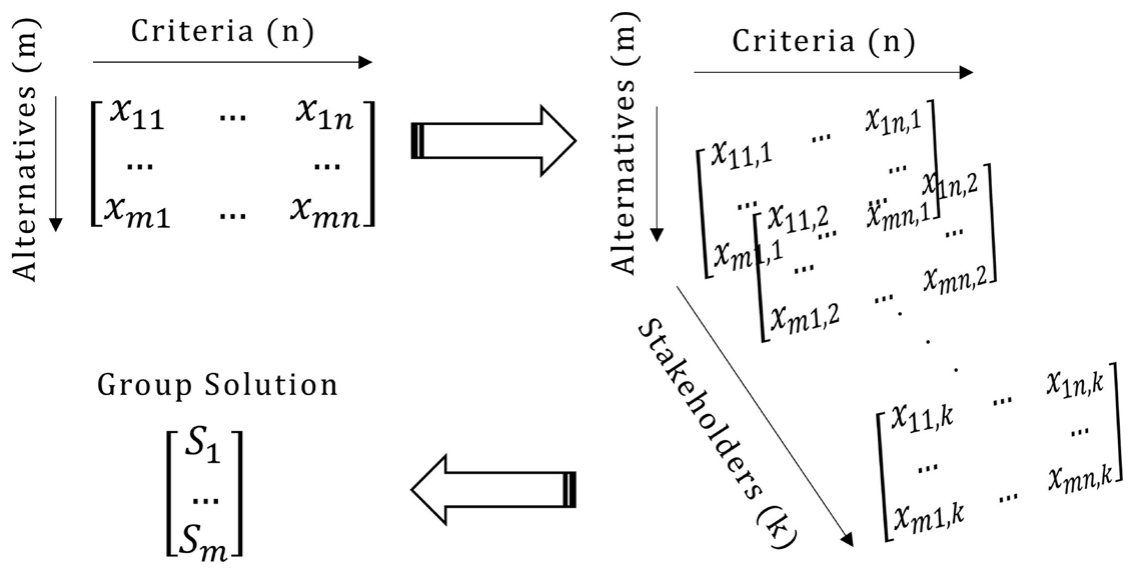
The group decision making process followed in APOLLO-Live.

The methodological foundations of the tool are rooted in the operations research domain, drawing in particular on the MCDA, GDM, and consensus making and reaching fields. It employs the 2-tuple group TOPSIS method, which combines TOPSIS ^39^, a well-established MCDA methodology for ranking alternatives, with 2-tuples ^40^ that essentially constitute an expansion of linguistic terms that are easy for stakeholders to comprehend, as well as with a GDM setup ^41^ in that it is based on multiple stakeholders voting simultaneously. In particular, the typical MCDA problem consists of two-dimensions with alternatives being evaluated against certain criteria. When employed in a GDM context and the stakeholders offer the evaluation, this problem is converted into a three-dimensional problem; in that case, the additional dimension consists of the multiple stakeholders participating in the process ( Figure 1). The aim is to calculate a ‘group solution’ combining all votes of the stakeholders and expresses the belief of the group as a whole.

To reach the group solution, the most common approach is to aggregate the *k* matrices presented in Figure 1 using weighted averages to configure a new decision matrix that represents the entire group. Then, after another set of weighted averages, the vector representing the final group solution can be calculated ^42^. Alternatively, and instead of calculating a group decision matrix, Krohling and Campanharo ^41^ calculated a vector representing the solution of each independent stakeholder using the fuzzy-TOPSIS method. Then, they combined these vectors in a new matrix, where this time the stakeholders essentially comprised a new set of evaluation criteria of the MCDA process, before eventually using another round of fuzzy-TOPSIS to calculate the final group solution. APOLLO-Live uses the latter approach, since in energy and climate policy it is important not only to reach a group solution but also to understand the conflicts existing in the individual solution of each stakeholder ^20^. It also employs the 2-tuple TOPSIS instead of the fuzzy TOPSIS ^34^, since the use of 2-tuples is usually closer to the way stakeholders operate—compared to fuzzy sets ^20^.

Additionally, by selecting only the stakeholders that belong to a certain category (e.g., based on socio-demographics, occupation, etc.), we can follow the same approach to calculate the solution of each category and compare them (e.g., identify how the results differ based on the viewpoint of academics compared to industry experts or policymakers). The rationale for performing these types of category-based analyses lies in the fact that the tool is envisaged to be used in workshops with participants from diverse backgrounds. However, in such cases, it is possible that markedly divergent preferences may be exhibited based on the background of each participant ^20, 21, 43^. Comparisons between the collective solution and the solutions preferred by members on a specific category may reveal patterns that advance the debates.

The methodological choice followed comes at a disadvantage with regards to consensus reaching processes. Such processes refer to feedback mechanisms that provide stakeholders with suggestions on how to willingly change their votes towards increasing the consensus levels of the group ^37^. Usually, in the first approach, which uses aggregations based on weighted averages, the group decision matrix and the matrices of the stakeholders are of equal dimensions and format, making them easy to compare and provide the required tips. This is not the case when first calculating the individual solution for each stakeholder and then the global group solution, due to the difference in the dimensions of the initial matrix that the stakeholders vote on and the matrices emerging along the way. To solve this, in APOLLO-Live we apply a consensus reaching process based on tips using the following protocol. We first use the consensus measurement metric introduced in ^34^, which compares the individual solution vector of each stakeholder with the group solution vector, and calculates how close each stakeholder is to the group solution, thereby offering a universal consensus of the group (expressed as a percentage). Then we identify the stakeholders that feature a proximity to the group consensus below the average of the group and find the alternatives with the highest distance between their solution and the group solution. Finally, in the subsequent voting rounds, stakeholders are asked if they want to change their votes on the identified alternative, offering changes to their evaluations against all potential criteria. Usually, in computer-simulated GDM and consensus reaching, a core aspect is the speed of convergence ^44^. However, in our case, convergence lies entirely in the participants’ behaviour, which is not always cooperative as implied in all simulated cases, where some form of compromise is always assumed ^42^. To facilitate this process, a target featuring a predefined desired level of consensus and a total number of voting rounds that can realistically be performed (e.g., to account for participants’ fatigue) should be agreed with the participants beforehand. In this case, the deliberations end once the first of the two conditions are met (i.e., the desired level of consensus is achieved, or the total number of voting rounds is reached without the desired consensus).

### Implementation

The APOLLO-Live webtool is a sophisticated platform designed to facilitate anonymous online voting workshops, while ensuring data security and integrity so that stakeholders and interested parties feel confident and safe to participate. This application leverages Hexagonal Architecture, allowing the application core to define abstract interfaces (ports) and leaving the implementation details (adapters) to be provided by external components. This architecture promotes the Dependency Inversion Principle (DIP) which enhances flexibility and testability, as the core remains decoupled from specific technologies or frameworks.

The clear separation of concerns and the use of interfaces make it easier to write unit tests for the application core. Test doubles can be easily created for ports, allowing for isolated testing without the need for external resources. In addition, the modular structure of Hexagonal Architecture facilitates the maintenance and future extension of the software. Changes to the external interfaces do not affect the core logic, reducing the risk of introducing bugs and enabling easier transitions between technologies and frameworks. Replacing or updating external dependencies is simplified, as long as the new adapters conform to the existing ports.

**Figure 2.**
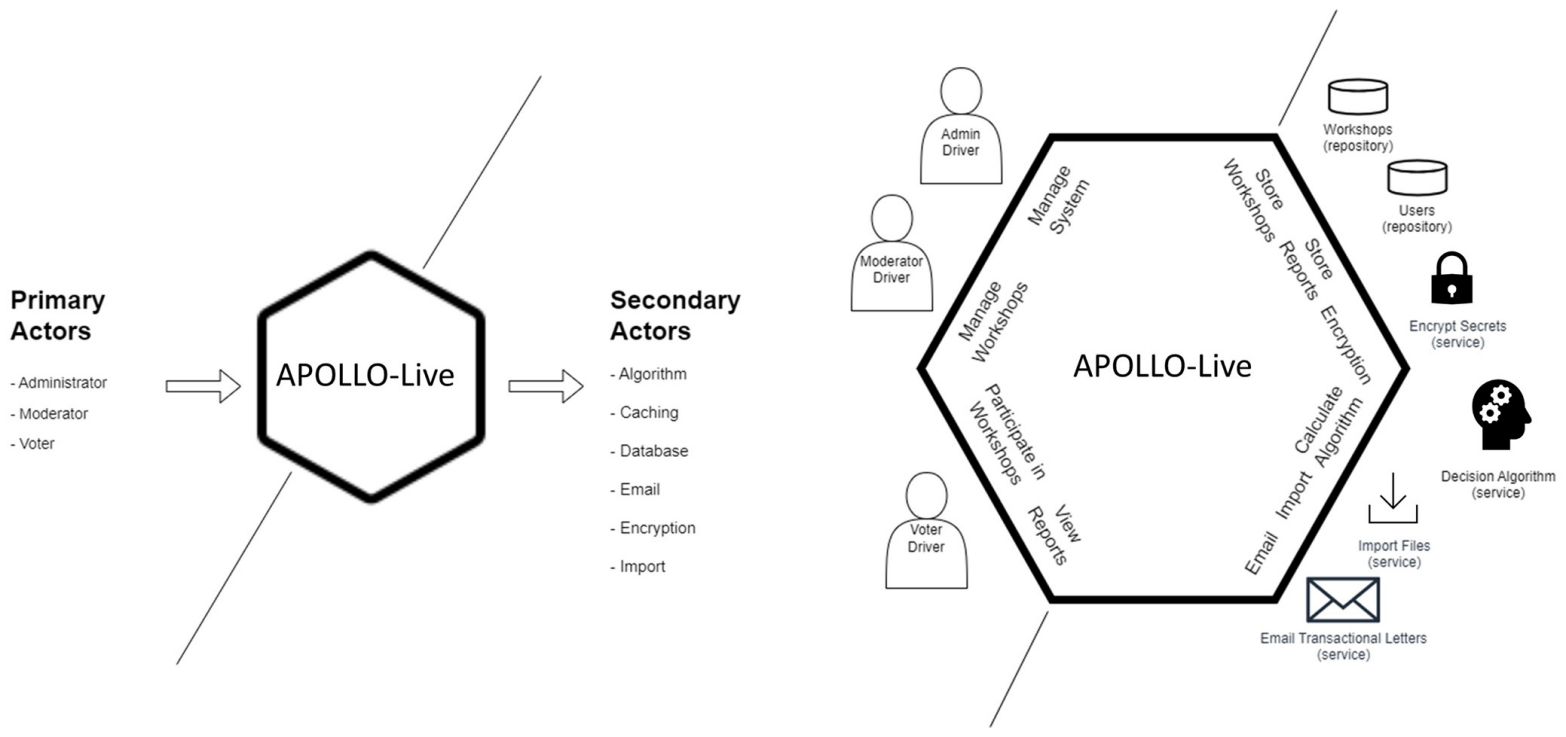
Primary and secondary actors including their respective ports in the Hexagonal Architecture of APOLLO-Live.

The first step when designing with the Hexagonal Architecture is to define the primary and secondary actors. In our case, we have three primary actors: the administrator of the system, the moderator of the workshops, and the voter(s). Our secondary actors are the algorithm used to support our decisions, the caching mechanism of the requests, the database, the emailing system, the encryption system, and the import methods ( Figure 2). The second step is to identify our ports by envisioning different interactions among the actors. These interactions are then implemented through ports, using a technology-agnostic programming interface. The primary and secondary ports are presented in Figure 2.

Finally, we define the adapters, following the principle that for every adapter we typically have two implementations: the “real” adapter doing the work in the production-level system, and the tester that tests the port’s functionality. In our case, we have the following adapters:

- Algorithm Adapter, which implements the 2-tuple TOPSIS calculations
- Database Adapter
- The Entity Framework, which supports all known databases like SQL Server, MySQL, MariaDB, PostgreSQL, SQLite—the current implementation works with a PostgreSQL database
- Cache Adapter, using .net core process in-memory caching
- Email Adapter, that sends e-mail messages using the SMTP protocol
- Encryption Adapter, using Entity Framework Core and Microsoft Data Protection stack to store secret keys
- Import Adapter, using Excel files to import manually-ran workshops
- UI Adapter
- .net core web app
- .net xunit acceptance tester.

In essence, by using Hexagonal Architecture and integrated with the 2-tuple Group TOPSIS algorithm, the APOLLO-Live webtool offers a secure, scalable, and decision-enhancing platform for anonymous online voting workshops. It seamlessly integrates cryptographic techniques, real-time communication, and multi-criteria decision-making to ensure a trusted and engaging voting experience.

### Operation

Considering that APOLLO-Live is intended to be used live in workshops, the operation of the tool can be viewed from two perspectives: (i) the participants of the workshops; and (ii) the organisers and by extension the moderator(s) of the workshop.

In both cases, the webtool is considered a lightweight application without intensive background processes. For potential organisers that intent to host the tool in their own servers (see Software Availability statement) the following minimum hardware requirements may apply (depending on the expected traffic):

General Requirements:

- CPU: 1 vCPU (1 GHz or higher)
- RAM: 512 MB – 1 GB
- Storage: 1 GB of available disk space (actual usage is typically lower but depends on data retention and usage patterns)

Containerised Deployment:

- Alpine-based Image: 512 MB – 1 GB RAM
- Ubuntu-based Image: 1 GB – 2 GB RAM

**Figure 3.**
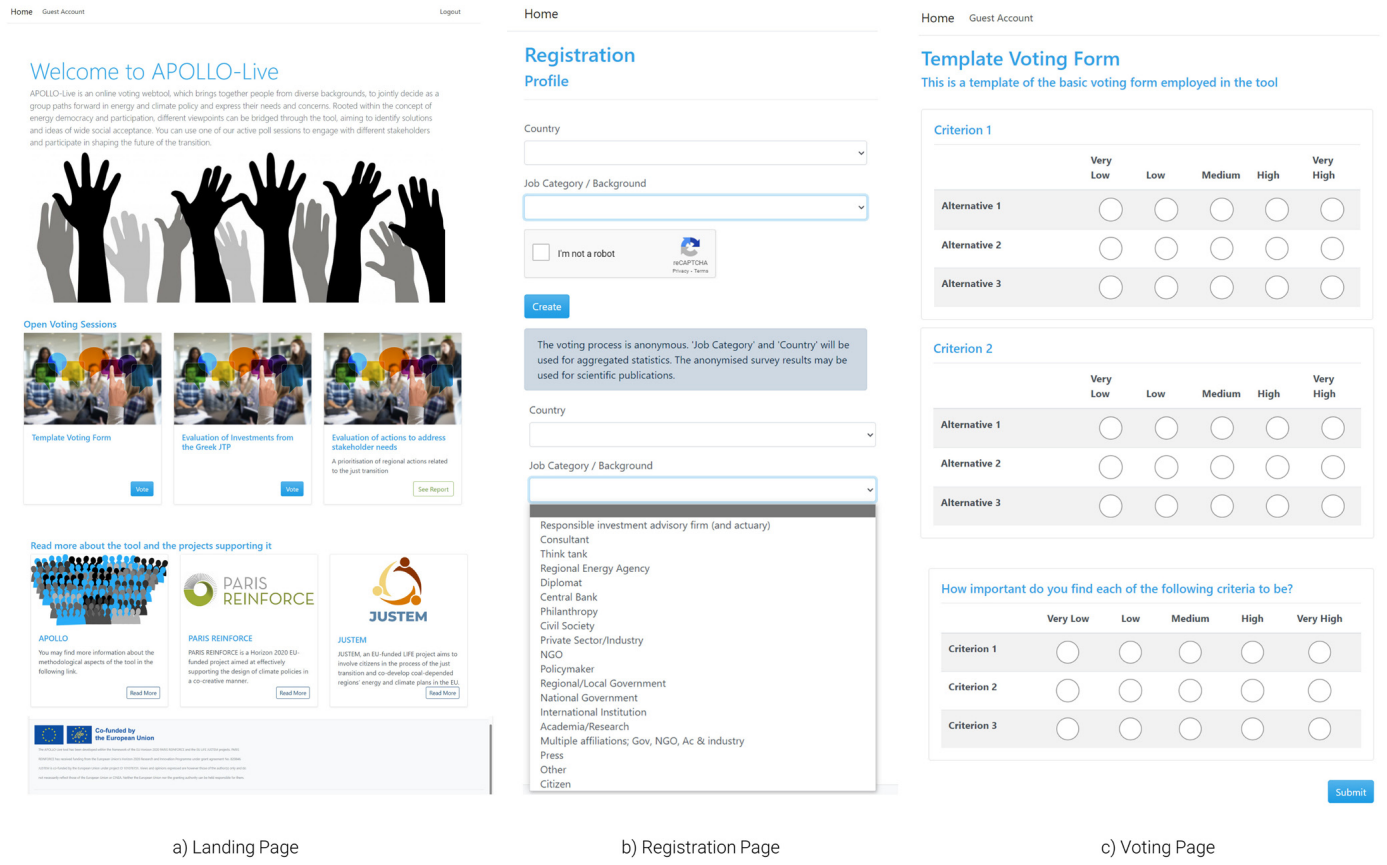
Initial pages of the APOLLO-Live webtool from the perspective of workshop participants.

***The tool from the perspective of the participants.*** Starting from the viewpoint of workshop participants ( Figure 3), users arriving on the landing page ( Figure 3a) can view information on the tool and choose one of the active poll sessions to participate in an engagement exercise with different stakeholders. It is also possible for users to land directly to the registration page if, for example, the organisers use QR codes to facilitate the process. After joining a poll, the user is initially asked to provide some basic information on the country and background of the users ( Figure 3b). This information is collected to facilitate the category-based analyses described in the methods section. The tool collects no names, e-mail addresses, or other information that can be used to identify the users. A disclaimer explicitly explains how the data collected (in reference to the user’s background and country) will be used to provide aggregated results. Through drop-down menus, the tool can facilitate a wide range of backgrounds, enabling engagement that is as inclusive as possible. Hence, the tool Additional background options and extra profile variables can be added in future iterations of the tool.

After providing the required information, the user is directed to the voting page of the selected session. In Figure 3c, a generic template of such a survey is presented. In real-life applications, both the alternatives and the criteria will be filled out by the organiser, while detailed information (e.g., questions) will be displayed to ease the voting process. Finally, the user can also provide values for the criteria weights—i.e., importance of each dimension—against which alternatives are evaluated. The uniqueness of these weights reflects the independent valuation and preference system of each participant.

**Figure 4.**
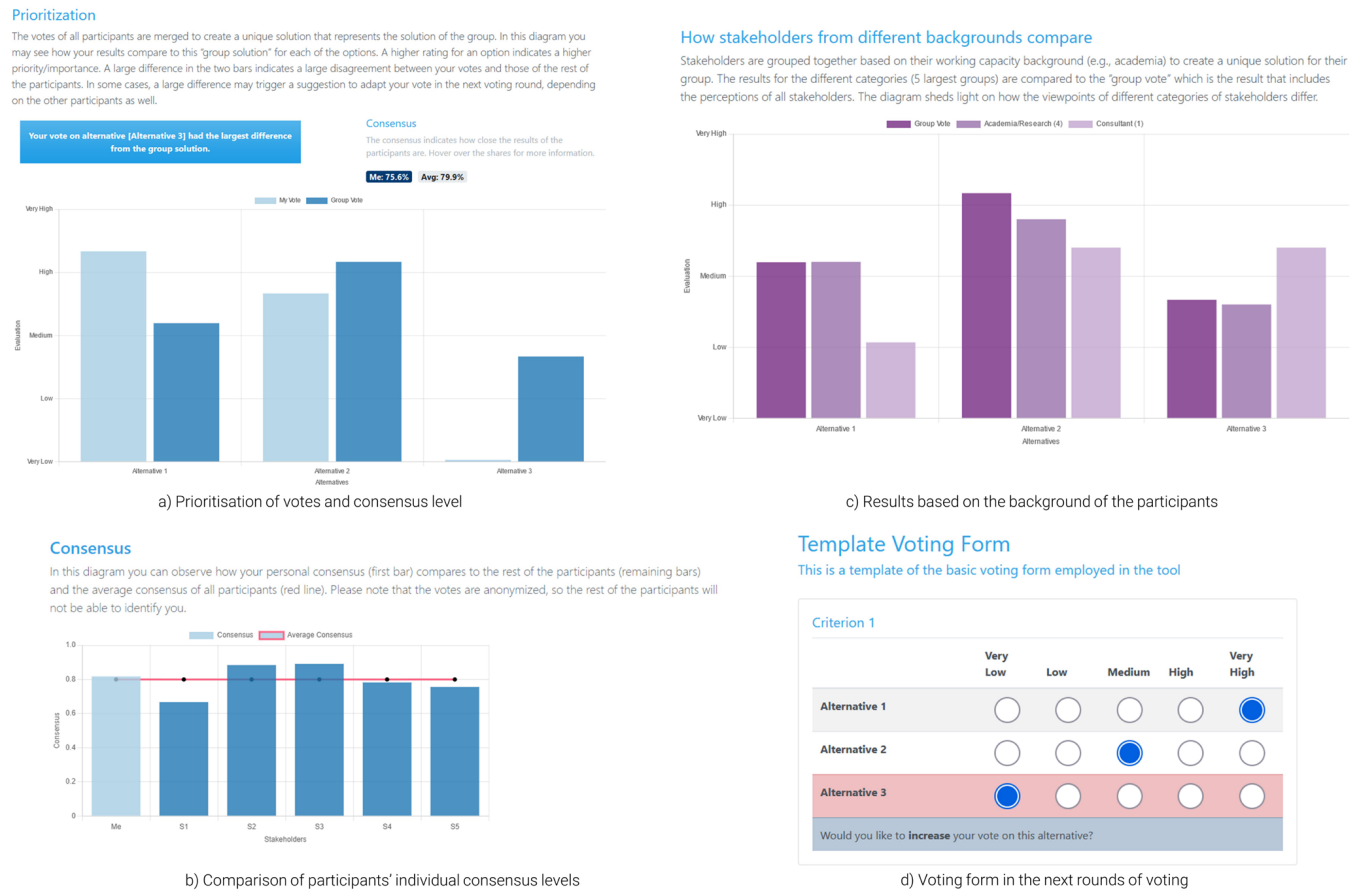
Results page and voting form for the follow-up round of voting.

During workshops, when all participants cast their votes, these are analysed in the background. While a user waits for either the results to be calculated or other participants to vote, they are presented with a loading page. Once the calculation process is over, the participants are automatically redirected to the results page ( Figure 4; the results presented in the figure are based on random responses). This marks the beginning of the feedback process, where results are discussed among participants and a moderator, across three dimensions: (i) prioritisation ( Figure 4a); (ii) consensus ( Figure 4a–b); and (iii) results based on the background of the participants ( Figure 4c). Essentially, the participants can see a rank of the alternatives that reflects the importance given to each option by the whole group, the consensus level, indicating how close the votes of the participants were and how each participant is positioned within the group. All participants are anonymised, meaning that a user can see the consensus level of the other anonymised users, but not any identifying attributes. Finally, the tool presents results based on the background of each user, to flesh out different dynamics between users of different working capacity. This attribute can be expanded to incorporate different groups (e.g., analysis based on gender, age, etc.). Each plot within this page is coupled with explanatory text to help the participants understand and interpret the results presented in the figures. As indicated in the overview of the theoretical concepts, participants with lower levels of consensus receive tips for adapting their preferences in the next voting rounds to improve consensus. These tips are provided during the presentation of the results ( Figure 4a) and the re-opening of the voting form with their previous votes pre-filled ( Figure 4d); the users are automatically redirected there by the moderator once discussions are over.

**Figure 5.**
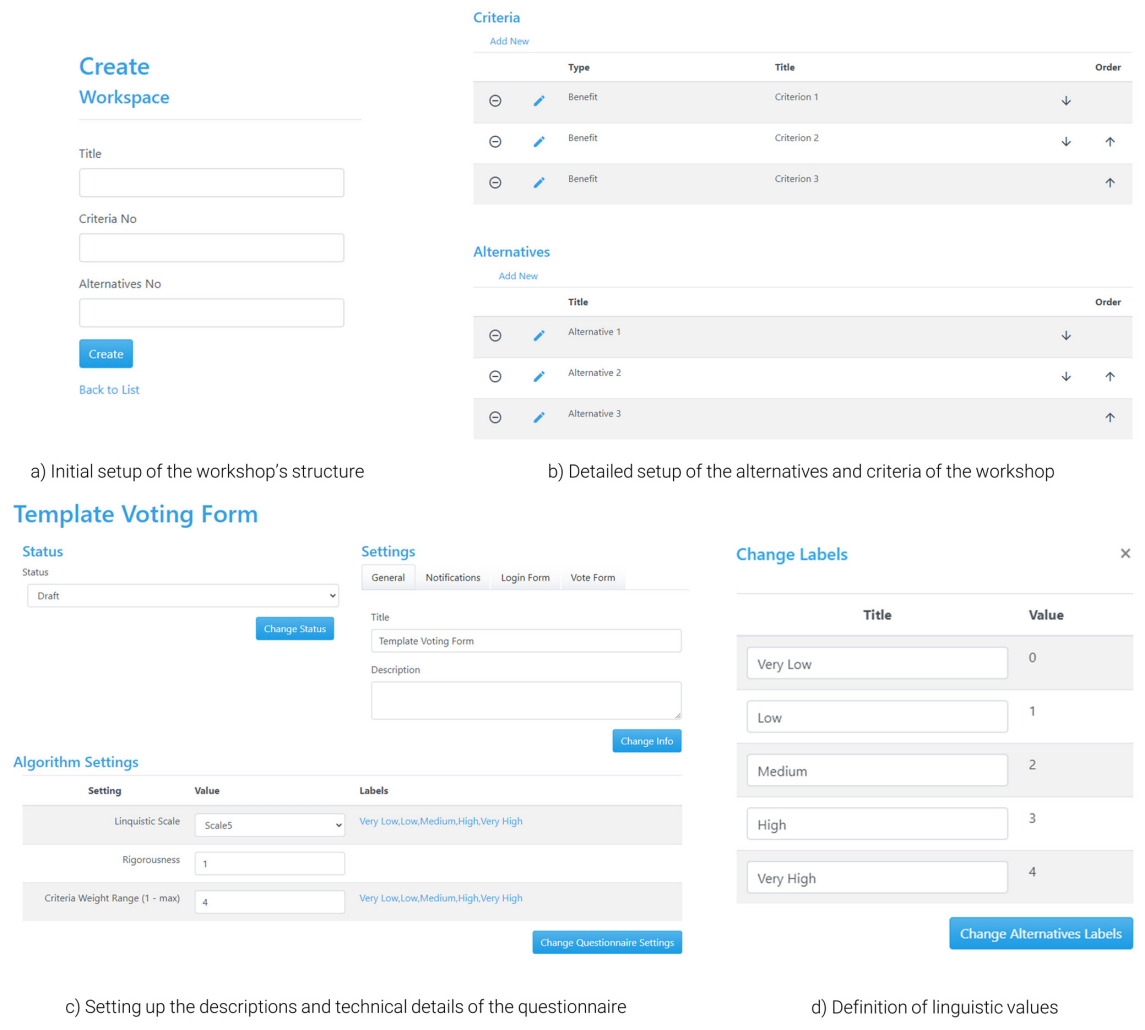
Main interface for setting up a questionnaire in APOLLO-Live from the perspective of the organiser.

***The tool from the perspective of the organisers.*** To allow organisers and moderators to facilitate the operation of the workshop, the tool includes a slightly differentiated interface ( Figure 5), but with additional functionalities to enable setting up the workshop’s structure and moderating the process (i.e., calculating results and managing the direction of users between the presentation of results and voting). Any interested party intending to host a workshop using the tool must register for an ‘admin’ account, which is an open and free process, but is subject to confirmation from the development team. Alternatively, the tool can be downloaded from GitHub (see software availability statement) and launched in the users’ server of choice. When logging in as an admin, an organiser has the chance to define the setup of a new workspace, which represents the activities they wish to perform during the workshop. When setting up the structure, the organiser adds basic information regarding the questionnaire of the exercise, such as the name of the workshop and the number of alternatives and criteria, which can also be adapted later ( Figure 5a).

In the main interface for defining the details of the structure of the questionnaire, there are two types of information that should be filled in ( Figure 5b–c). The first type refers to the presentation of information to the participants so that they have a proper understanding of what is required from them; these include details on the name and description of the questionnaire, which are presented both in the landing and the voting page and could help users choose the poll they are interested in (e.g., in the case of parallel polls), as well as understand how to fill in the survey. The second type is related to technical information, including the set of parameters necessary for the framework of the tool to be operational based on the defined theoretical principles; such information includes the choice of linguistic terms used for evaluating the alternatives and the criteria (e.g., number of scales, qualitative terms, and their interpretation to semi-quantitative values; Figure 5d), which can be similar for the two cases depending on the overarching structure of the questionnaire. Finally, the 2-tuple Group TOPSIS method requires setting up a rigorousness parameter, which depends on the level of ‘penalty’ that should be imposed to disagreements in the calculation of the consensus (i.e., same level of disagreement leads to larger reduction in consensus under a strict parameter). This value is suggested to be set between 0.6 (strict value) and 1 (usually employed when there is only one round of voting), with the middle value (0.8) usually being employed when multiple rounds of voting are expected ^45^.

Once the structure is set, a workshop moderator —who is not necessarily the organiser, although the two should be in close collaboration—can make the poll public so that participants can join and start voting.

**Figure 6.**
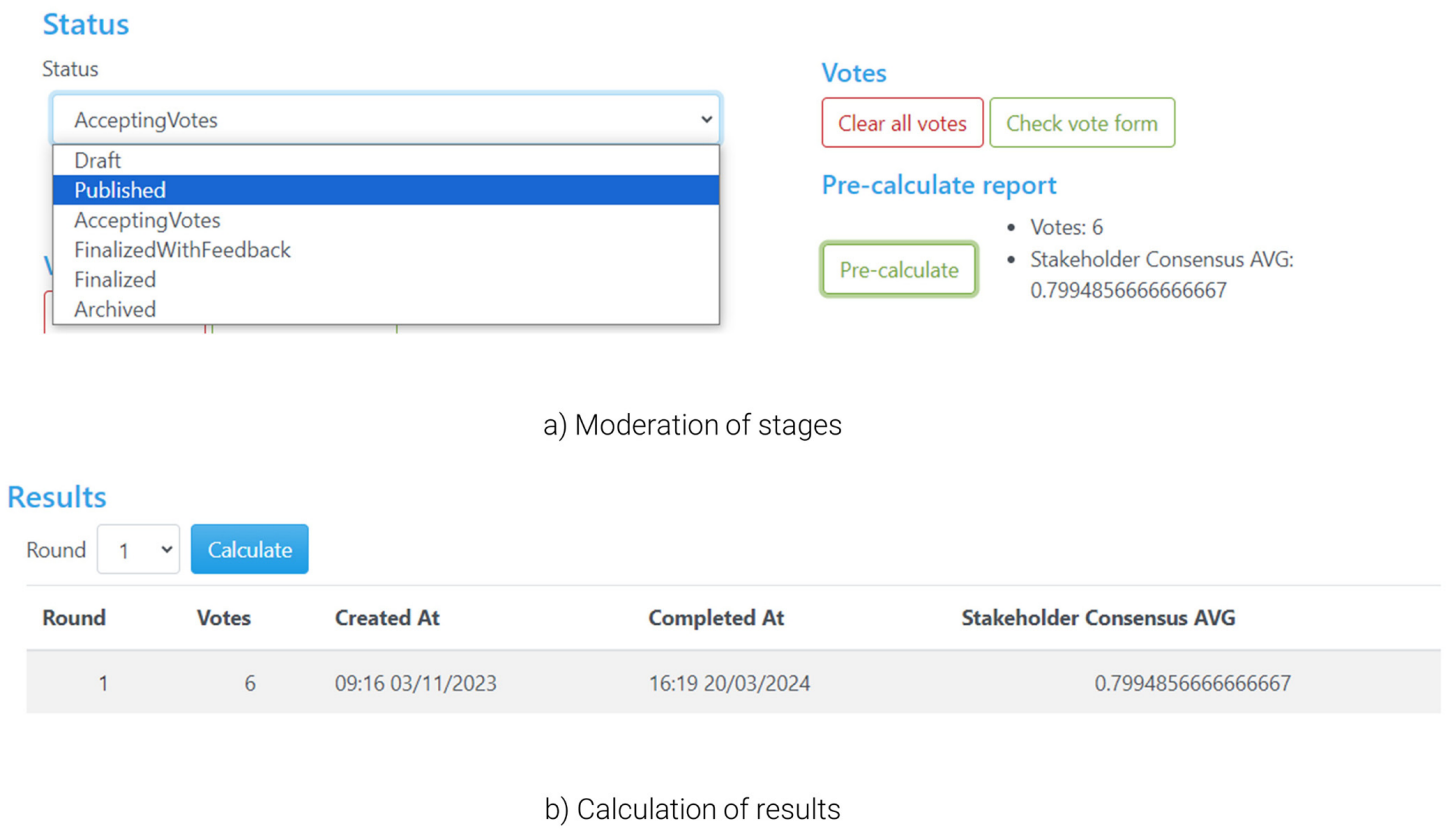
Stages of the voting process and calculation of results.

During the preparation of the workshop ( Figure 6), the admin should set the status ( Figure 6a) to ‘Draft’ in order to be able to configure the questionnaire, such as defining the alternatives and criteria in detail. During the ‘Draft’ stage, the poll is hidden from non-admin users. Once the setup of the questionnaire is finalised, the admin can change the status to ‘Accepting Votes’, which creates a public poll in the land page that users can access and start voting (as in Figure 3). During voting, the tool offers the organiser/moderator some functionalities to facilitate the voting process and have a better overview of the flow of the workshop, notably including a ‘pre-calculate’ button that monitors the number of participants that have already submitted their responses, the current level of consensus, or options for the moderator to send in-tool messages to participants (e.g., in case there are time limits for voting). Once all participants have voted, the admin can proceed with the next steps through the ‘Finalised with Feedback’ status, which closes the poll (no further voting is accepted onwards). This allows the admin to calculate the solution ( Figure 6b) and automatically redirects users to the visualisation of the calculated results, including the tips highlighted in the previous sections.

During the ‘Finalised with Feedback’ status the users have access to the voting results (including their own results and the entire group’s), and hence this is the primary stage where deliberations among participants take place. By sharing their screen and the group’s results, the moderator can help all participants follow these discussions closely. No personalised results are presented as the moderator is not voting; these can be viewed by each user on their own screens as well. Once discussions are concluded or reach a stage at which there are indications that a follow-up vote can lead to improvements in terms of resolving conflicts within the group, the moderator can change the status to ‘Accepting votes’ again, which provides the participants with the opportunity to change their votes.

**Figure 7.**
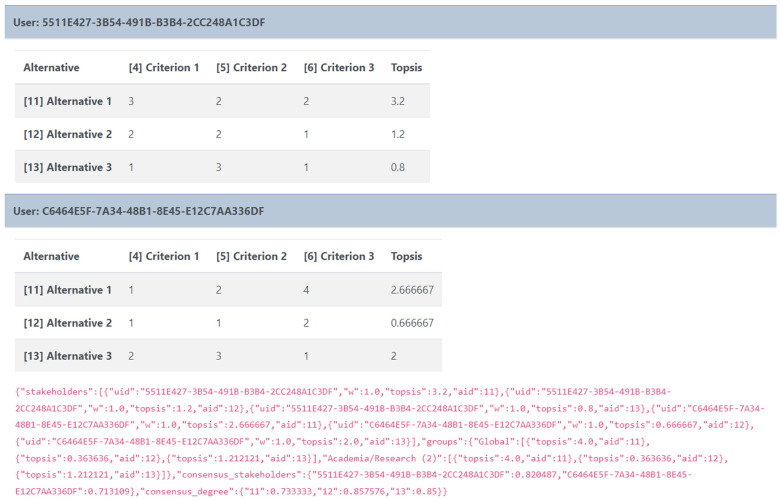
Data format for the results of each workspace.

Once the workshop is over, the admin can view and download the results either for further processing or archiving offline in a concise JSON format ( Figure 7), which is easy to handle by most common data processing software and ensures anonymity as it maintains the encrypted user-ID used within the tool.

The final functionality offered to admins and workshop organisers is that of an asynchronous analysis, which is essentially a solver of the 2-tuple Group TOPSIS without the feedback mechanism for consensus reaching, since in this setup no follow-up voting can occur. Although the tool is primarily developed for synchronous use in workshops, occasionally polls are rolled out in advance of a workshop or are used as a completely standalone engagement process. In this case, the “Import” functionality in the workspace section of the tool enables an admin to upload an excel with the format of the workshop and the responses of the stakeholders, with the tool then creating a workspace, compiling the votes and immediately offering the opportunity to calculate the results based on the 2-tuple Group TOPSIS previously described. The template to do so can be found in the supplementary material and the tool’s GitHub page (see Data and software availability section), and it closely resembles the format produced by common online forms (e.g., Google forms) to increase interoperability.

## Use cases

In this section, we will present two use cases of the tool to elicit stakeholder opinions on aspects of climate action and just transition with emphasis on the role and perspectives of the youth. The two use cases represent the two main functionalities of the tool presented in the methods section: the synchronous (live) use of the tool in workshops, and the asynchronous analysis on previously conducted polls. Although intended to constitute concrete examples of how the tool can be used, the two use cases should be primarily treated as pilot appraisals with numerical examples showcasing the capabilities of APOLLO-Live, rather than concluded research with policy-relevant outputs; the group of participants do not necessarily represent representative samples of any particular stakeholder group, neither the selection of alternatives and criteria can be viewed as potentially exhaustive or complete.

### Use case I (synchronous use): Live evaluation of actions the youth can undertake to support climate action

In this first use case, we attempted to collect the perceptions of undergraduate students among those enrolled in the climate change mitigation modelling course of the School of Electrical and Computer Engineering, at the National Technical University of Athens, with regard to a diversity of actions young scholars can undertake to support climate efforts. Participation was voluntary—with no academic incentives provided—and anonymous (for the voting stage) as clearly communicated to the participants in advance and through disclaimers within the tool itself. Although anonymity within the tool is de facto ensured by software design (i.e., neither the participants nor the organiser can identify the origin of any particular vote), expectedly as with any live workshop the discussions can provide hints on the viewpoints of specific participants, which is however expected during such deliberations. Five students showed interest and participated in this exercise.

The selection of alternatives, criteria, and linguistic scales used to elicit the participants perceptions are presented in Table 1. The overarching theme of the questionnaire focused on what young academics should do to advance climate action and the role of science in society. Hence, a good alternative out of the five possible ones within this configuration would be one that has a large impact on climate action, increases the trust of the public in climate science, and improves the quality of science itself.

**Table 1.** Configuration of alternatives, criteria, and linguistic scales for the first use case.

		**Criteria**		
		Impact on climate action	Legitimacy of climate science as perceived by the general public	Quality of climate science
**Alternatives**	Pursue positions in the government	Linguistic Scale: {Very Low, Low, Medium, High, Very High} *Example: “What it the impact on climate action of pursuing positions* * in the government?*		
	Participate in climate activism			
	Improve communication skills			
	Seek collaborative projects with the private sector			
	Adopt climate-conscious behaviours			
	**Criteria Weights**	Linguistic Scale: {Very Little, Little, Moderate, Much, Very Much} *Example: “How important do you consider the impact on climate* * action when evaluating a potential action?*		

Two rounds of voting took place, while no predefined target for the level of consensus was established due to time restrictions. Initially, the organisers (authoring team) provided a brief context on the questionnaire and what is expected from the participants regarding the voting process, avoiding detailed explanations to ensure that the participants will not be biased. Then, the participants accessed the tool and started voting. After the first round, the results were calculated and presented, including the consensus metrics and the sources of deviation from the group’s aggregate results. At this stage, a discussion took place between the participants with the facilitation of a moderator; when this process was over, the participants started voting again, this time receiving the feedback from the tool and the discussion itself. Eventually, the final results were presented and discussed.

**Figure 8.**
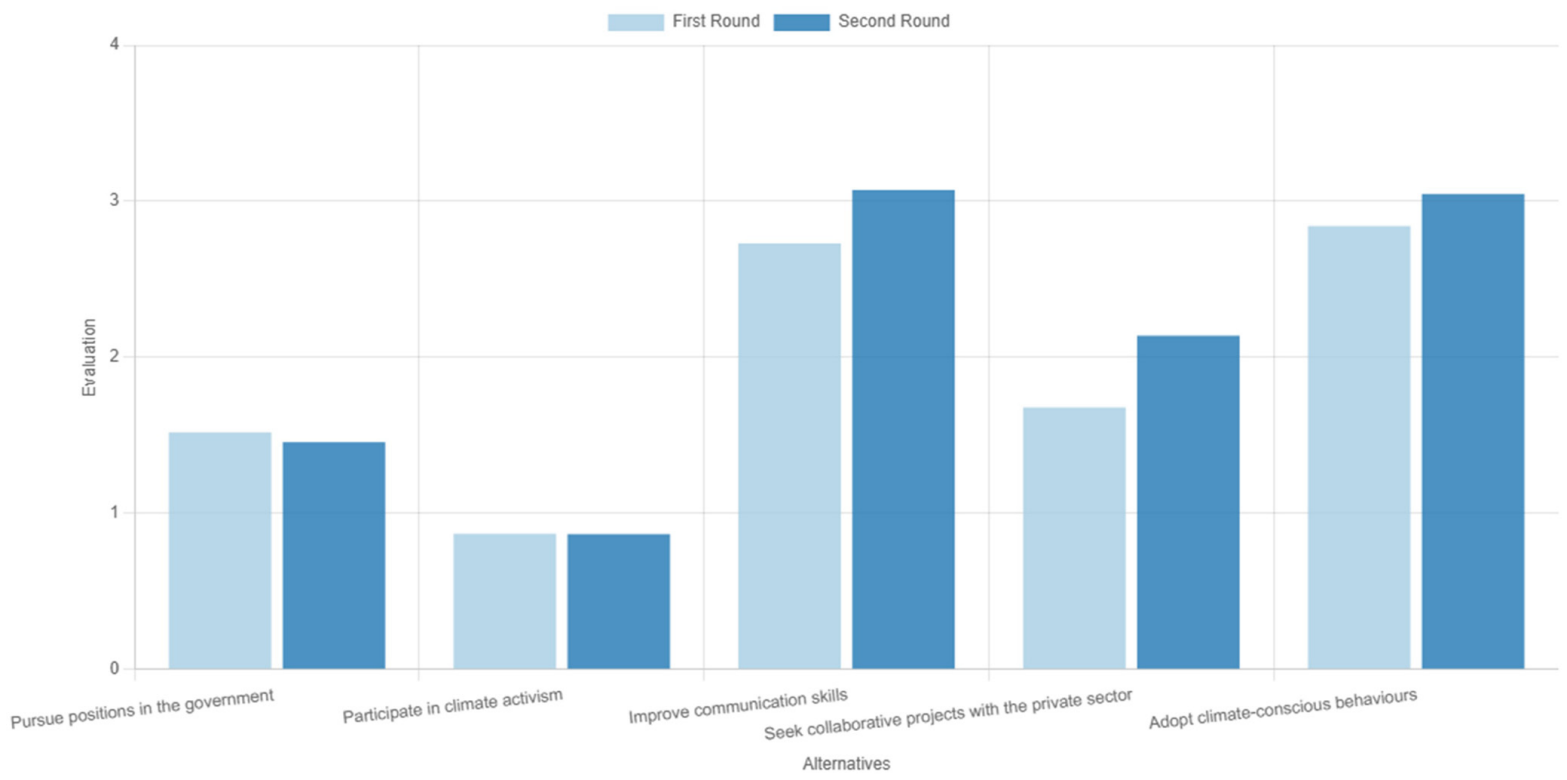
Ranking of the alternatives in the two rounds of voting during the first use case.

The results from the two rounds of voting are presented in Figure 8. We observe that two alternatives were the most dominant: ‘improvements on communication skills’ ranking marginally first, and ‘adoption of climate-conscious behaviours’. Then came ‘collaboration with the private sector’, with the other two alternatives following at a distance. Consensus was measured at 78.5%, an overall average result, but adequate considering that only two rounds of voting were performed.

These results generally reflect two interesting discussions among the participants during the deliberation stage in between the voting rounds.

The first discussion focused on the proximity of the two alternatives that were featured in the first places of the ranking order. Already during the first round, these two options were considered as top actions compared to the rest, but in a reverse order—i.e., adopting climate-conscious behaviours was the preferred alternative against the selected criteria. However, some participants stressed that, although leading by example is good practice, improving communication skills has the potential to impact a larger audience and for a longer time. A discussion followed on the impact of these two, which evidently caused a shift in the group’s sentiment regarding the two alternatives; hence, in the second round, the order between the two was reversed. Despite the marginal differences, this example underpins the added value of deliberation as a process that may gradually shift opinions, but also of the tool itself, as in ranking methods the order can be deduced by small margins ^42^: even marginal changes in ranking scores could have important implications for a real-world problem, where a winner-takes-it-all solution is sought or where a ranking of alternatives can influence the synthesis of an investment portfolio.

The second discussion also featured a debate on two alternatives. This time, the discussion focussed on the ‘pursuing government jobs’ on the one hand and ‘collaborating with the private sector’ on the other. The participants discussed the pros and cons of each option, with the outcome of this discussion not leading to any change in the order of the alternatives, like before, but instead strengthening the gap (score difference between the two) emerging after the first voting round.

The participation in climate activism actions ranked in the bottom, despite youth movements gaining traction as a means of protest ^46^, with evidence showing such initiatives are overall viewed positively by the youth ^47^. This outcome may be interpreted through multiple lens, based on discussions with the participants at the end of the process. First, even among the young generation, there may be largely different opinions on the effectiveness of a certain action; this may reflect the opinion of only a small sample, but it nonetheless attests to the need to significantly expand deliberation and co-creation activities to include the youth and capture a broader viewpoint of this stakeholder group. Second, the actions provided to the participants as options are not mutually exclusive, meaning that results merely reflect prioritisation rather than selection. Considering the small sample caveat, these elaborations act as an example of how outcomes of the tool should be dynamically interpreted through active discussions with the participants, instead of strictly based on the quantitative results.

### Use case II (asynchronous use): Ex-post analysis of the youth’s elicited perspective on actions for a just transition

Within the second use case, we shift the focus to the just transition of coal-dependent regions—similarly to the first case, however, through the lens of the youth again. This case featured an asynchronous analysis of polling results, outside the scope of a workshop, and serves to highlight the complete range of functionalities of the tool and its interoperability with other voting platforms. Notably, a Google Form was set up to approach young people (<25 years old) in Western Macedonia, Greece, the country’s primary coal-producing and -dependent region, and thus the most vulnerable to energy transition policies and to the impacts of Greece’s coal phaseout ^48^. The voting form was also completely anonymous (only gender information was collected for aggregate analyses that cannot be traced back to the voter), with the form providing relevant disclaimers. 13 participants filled out the survey.

The alternatives, criteria, and employed linguistic scales are presented in Table 2. The alternatives constitute mixes of potential actions that the national and regional governments could pursue to mitigate the impacts of coal phaseout in the region. The selection was based on the actions outlined in the territorial just transition plan of Greece ^49^, thematically grouped to simplify the exercise given that the organising team could not provide elaborations. Therefore, further information such as which actions these policy mixes could entail were provided to the participants in the voting form. Given the age focus of the target group, relevant criteria were selected, relating to potential impacts of the actions on the region, today’s youth future in it, the participants’ peers, and the entire regional community. Contrary to the first use case, we could not ask the participants to share their views on the contribution of each criterion to their personal preference model, considering the asynchronous nature of the questionnaire.

**Table 2.** Configuration of alternatives, criteria, and linguistic scales for the second asynchronous use case.

		**Criteria**		
		How strong is the ability of the following actions to improve conditions for you and your future in the region?	How strong is the ability of the following actions to improve conditions for your peers and their future in the region?	How strong is the ability of the following actions to improve conditions for other members of your community in the region?
**Alternatives**	Reskilling programs	Linguistic Scale: {Very Weak, Weak, Moderate, Strong, Very Strong}		
	Research Infrastructure and Innovation Valleys			
	Energy or Industrial Infrastructure			
	Supporting Development Actions			
	**Criteria Weights**	Fixed equal weights		

**Figure 9.**
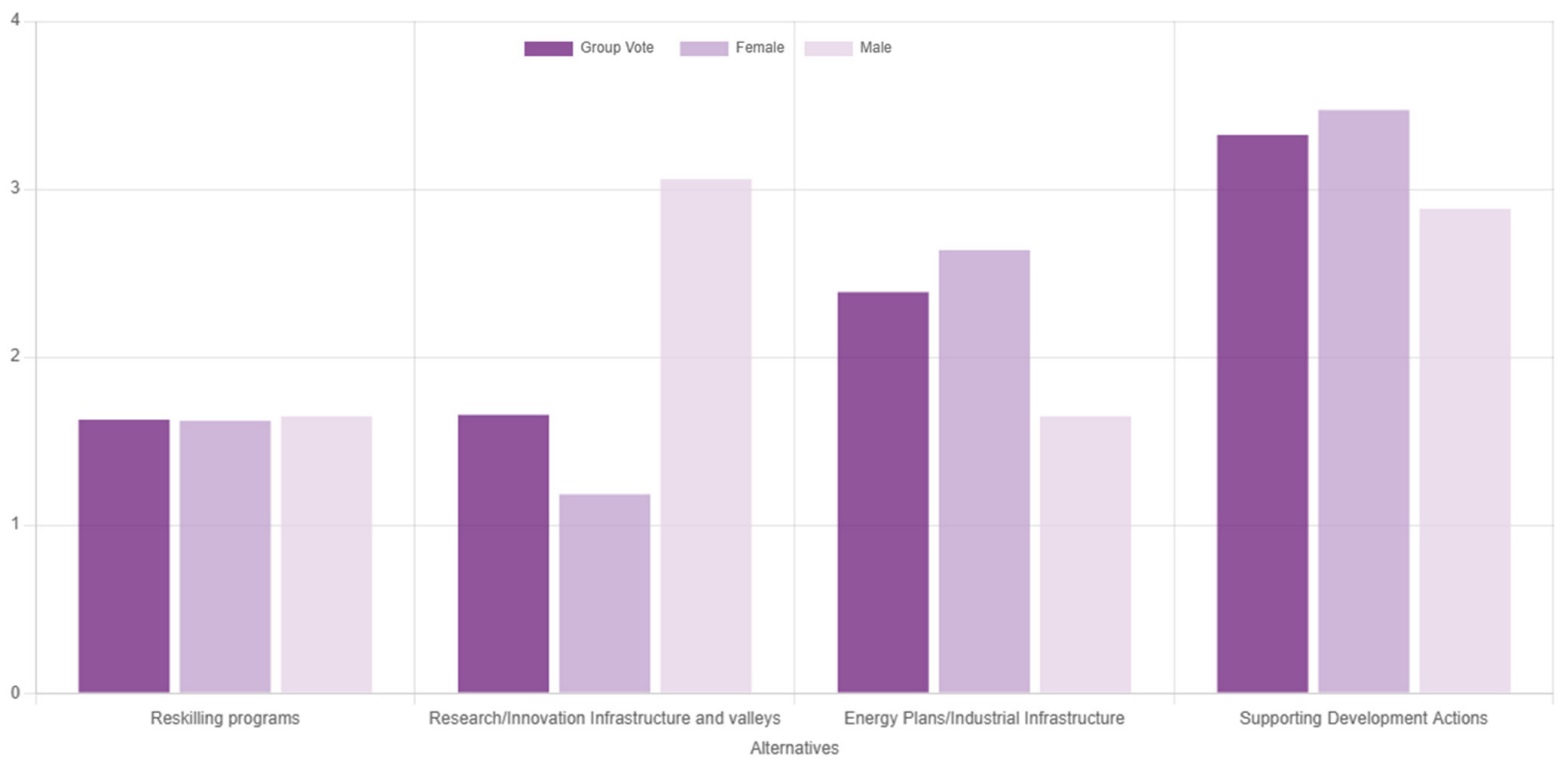
Ranking of the alternatives with gender-based disaggregated results during the second use case.

Figure 9 presents the results of the second use case. Evidently, supporting development actions was deemed as potentially the most effective route to mitigate regional socioeconomic impacts of the transition. Considering that coal phaseout can be viewed as a drop in regional competitive advantage ^50^, coal-dependent regions typically require a wide set of supporting actions to ensure their economic development is secured, which can explain this result. Following that, participants emphasised energy and industrial infrastructure as their second preferred action, potentially motivated by the already established energy-oriented identity of the region, with all other options following at a distance.

The consensus in this exercise was measured at 70.6%, which is relatively low and underpins the need for deliberation and consensus reaching processes through multiple rounds of voting, such as in the first use case. The low consensus level can be partially attributed to significant deviations of responders based on their gender, which is an interesting output on its own, given the underrepresentation of women in scientific processes and deliberations on climate change ^51^. Notably, female participants ranked ‘supporting actions’ first, while male participants prioritised the ‘research and innovation’ action instead, providing indications of the different needs the two groups face along the ongoing transition. Given the small sample that was relatively skewed towards female responders, not much should be read in these results, which should rather be used to reflect that the category-based analysis offered in the tool (i.e., results by group) can flesh out and potentially bridge areas of conflict between different viewpoints, while at the same time enhancing inclusivity.

## Conclusions

In this paper, we presented APOLLO-Live, a webtool to synchronously facilitate stakeholder engagement activities in workshops focusing on issues and debates arising in the energy and climate policy domain. With its methodological foundations rooted in group decision making and consensus reaching processes, the tool is capable of identifying common solutions within a group of participants, shedding light on conflicts among the different viewpoints expressed within the group, as well as providing relevant feedback to bridge said conflicts and calculating consensual solutions in public debates.

We also demonstrated its functionalities in two indicative use cases focusing on the young generation, one identifying preferred actions of young scholars to boost the impact of climate action and science, and the second evaluating actions related to the just transition of coal-dependent regions. Within these cases, we presented tangible examples of how the tool can be used to moderate discussions that shift the opinion of participants towards consensual solutions that might differ to their pre-existing beliefs, as well as flesh out different opinions based on the background of the voters.

A key strength of the tool lies in its flexibility, as it can be used to provide answers to a diverse range of questions. Essentially the tool is able to contribute, through a live stakeholder engagement process, to general topics that have so far been addressed by the use of asynchronous decision support tools and multi-criteria decision aid in energy and climate policy ^23, 52^, with the selection of alternatives, criteria, and linguistic terms being completely unrestricted. However, this also acts as a limitation of the tool, since the potential questionnaires that can be developed need to follow the common structure of MCDA problems, compared to online survey tools that incorporate multiple options for eliciting feedback, but lacking the functionalities of APOLLO-Live. Additionally, although when designing and developing the tool, our guiding principles focused on how it could be applied to problems related to energy and climate policy, this does not exclude potential uses of the tool across completely different domains, again with the caveat that following the standard MCDA format in designing future use cases is necessary.

Other limitations of the tool lie in the difficulty to use symmetrical scales with negative values, or handle responses such as ‘not able to respond’. On the former caveat, the method itself does not restrict the use of negative scales. However, it is possible for negative results to appear even in cases that the responder provided non-negative results (and vice versa), since TOPSIS is a distance-based method that can stretch results within the whole spectrum of the scale. This issue is significantly mitigated in scales of the same sign, and their use is advised. On the latter caveat, although efforts in the MCDA community have been made to introduce ‘not able to respond’ responses in MCDA exercises ^16^, the ability to actually implement such options depends on multiple conditions (i.e., notably the number of responders and in particular the requirement for a large sample in order to be able to replace the “not able to respond” responses with the average of the remaining participants), while in a tool used live in workshops there is no guarantee that these conditions are met. At the same time, the use of ‘do not know’ or ‘not able to respond’ has been contested in stakeholder engagement, as it may discourage people from responding in case of inter alia ambivalence ^53^. As such, to mitigate this caveat, it is suggested that the questionnaires are structured with clarity to avoid ambiguities and select a sample that is suitable to respond to the questions posed.

Based on these, future work on the tool includes its use in full-scale experiments with a large number of participants, tests across different domains and questionnaires, as well as incorporation of different solving techniques (e.g., MCDA methods other than the 2-tuple Group TOPSIS used in GDM ^54^), and consensus reaching mechanisms.

## Ethics and consent statement

Our study involved anonymous online surveys focused on perceptions on the energy transition—an area where ethical considerations per national standards and practices orient towards transparency, confidentiality, voluntary participation, and the right to withdraw, rather than a formalized institutional review board process as required in cases involving medical studies, clinical trials, or sensitive personal data. Researchers continuously ensured compliance with these considerations.

Notably, survey respondents provided the research team with their implied informed consent by completion of the survey for their participation and the use of the information shared in this study. The polls used included dedicated and transparent disclaimers informing respondents that their anonymous aggregated responses would be used for research purposes and published in a scientific article. Consent to using and publishing this data was inferred by completion of the survey. The data was collected under the assurance of anonymity, while respondents also retained the right to withdraw their participation and information at any point. All participants were over the age of 18. The entire engagement and elicitation process was conducted in compliance with ethical approval from the HOLISTIC Management Board.

## Data Availability

Repository: The APOLLO-Live synchronous GDM-CRP webtool: Datasets and Template https://doi.org/10.5281/zenodo.14510524 ^55^. This project contains the following underlying data: First use case data. Includes the data produced by the tool related to the first use case. Second use case data. Includes the data produced by the tool related to the second use case. Template. A file that can be used as input for an asynchronous analysis with APOLLO-Live based on the import function. **Data are available under the terms of the Creative Commons Attribution 4.0 International license (CC-BY 4.0).**

## Software availability

APOLLO-live is publicly available at https://apollo-live.epu.ntua.gr. Visitors that intend to try the deployed tool as workshop organisers should first register a new account which will be then approved by the tool developers. Users can also deploy the tool to a server of their own choice and run it independently.

- Source code available from: https://github.com/i2amparis/APOLLO-Live
- Archived software available from: https://zenodo.org/records/14851912
- License: The software is provided under the Apache-2.0 license (OSI approved open license).
