# Supplementary material for: APOLLO-Live: A multi-criteria-based webtool for synchronous group decision making and consensus support in energy and climate policy deliberations
Source: Open Res Eur. 2025 Aug 15;5:88. Originally published 2025 Mar 31. [Version 3] doi: 10.12688/openreseurope.19614.3 (PMC12280870; doi:10.12688/openreseurope.19614.3)
Supplement: Peer review history

# Reviewer report: Reviewer response for version 3

**DOI:** 10.21956/openreseurope.22907.r58333

**Published:** 2025-08-19

**Authors:** Phoebe Koundouri

**Affiliations:** 1 Technical University of Denmark, Lyngby, Denmark

I am happy with the revised version of the paper. The authors have successfully answered to my comments, clarifying the aspects that I raised.

Is the rationale for developing the new software tool clearly explained?

No

Is the description of the software tool technically sound?

Yes

Are sufficient details of the code, methods and analysis (if applicable) provided to allow replication of the software development and its use by others?

Yes

Is sufficient information provided to allow interpretation of the expected output datasets and any results generated using the tool?

Yes

Reviewer Expertise:

Environmental & Natural Resource Economics Sustainable Development Water & Marine Resources Energy & Green Transition. Climate Economics – Climate risk, carbon pricing, policy impact. Behavioral & Experimental Economics Interdisciplinary Systems Research

I confirm that I have read this submission and believe that I have an appropriate level of expertise to confirm that it is of an acceptable scientific standard.

# Reviewer report: Reviewer response for version 3

**DOI:** 10.21956/openreseurope.22907.r58332

**Published:** 2025-08-18

**Authors:** George Tsihrintzis

**Affiliations:** 1 University of Piraeus, Piraeus, Greece

My previous suggestions have been sufficiently addressed and responded to. I recommend acceptance.

Is the rationale for developing the new software tool clearly explained?

Yes

Is the description of the software tool technically sound?

Yes

Are sufficient details of the code, methods and analysis (if applicable) provided to allow replication of the software development and its use by others?

Yes

Is sufficient information provided to allow interpretation of the expected output datasets and any results generated using the tool?

Yes

Reviewer Expertise:

Artificial Intelligence, Decision Support Systems, Machine Learning, Generative Artificial Intelligence, Explainable Artificial Intelligence

I confirm that I have read this submission and believe that I have an appropriate level of expertise to confirm that it is of an acceptable scientific standard.

# Reviewer report: Reviewer response for version 2

**DOI:** 10.21956/openreseurope.22598.r56688

**Published:** 2025-08-04

**Authors:** Nebiyu Girgibo

**Affiliations:** 1 University of Vaasa, Vaasa, Finland

**Article title**: “APOLLO-Live: A multi-criteria-based webtool for synchronous group decision making and consensus support in energy and climate policy deliberations”

**Journal**: Open Research Europe

**Author**: Koasidis et al.

**General comments**

- It is a good article, and it is written well. A lot of pages for an article, but it is well if it is accepted by the Journal.
- If the minor comments are addressed for this article, I recommended it will be accepted based on editor’s decision.

**The article language check-ups and its editing**

 - The language is ok, well written and not seen that much language mistakes.

**Title**

- The title is great and it’s on the number of counts is ok, which is 15-word count. The maximum number of words shall be less than 12 and more than 6.
- Otherwise, the title represents the article text well even if it is a bit long one.
- It reflects the content of the article and its strength.

**Abstract**

- The abstract is good and it looks good, well organized and written with intellect.
- The abstract on well written and well reflecting the chance they have to show their passion in research of this kind.
- Please highlight the aims, novelties, contributions and significance of the study.
- Avoid using references in the abstract section.

**Keywords**

- The 7 keywords, which are the number of counts of key words that are in the requirement limit.

**The introduction**

- The introduction is short, it ok, well written and good level compressive that will help reader understand the concept.
-  It has a very good acceptability by the readers and help to understand the context of the article well.
- It is very ok, but the method used and the compressive paper is good.
- Research question should be included.
- A good level of references was used. The number of references can help understand well the climate change issue along with energy security.

**Methods**

- The methods written in was long and good one, well done.
- The method used was good, well-organised and impressive well to highlight the necessity of this method to work on this paper.
- In addition, the number of references and method, which is efficient are able to give a soon compressive review paper.

**Conclusions **

- The paper is good and it summarizes all the necessary section the article.
- The conclusion is very good, it is well written text, and good conclusions mentioned.
- It is matured and well written text that helps the reader understand the whole text is perfect.
- It has constructive conclusion that very goodly highlighted and showed the paper.
- Future direction section is quite well written.

**Abbreviations and Acknowledgments**

- Please include acknowledgment and abbreviation full list on your paper. If it is included, helps significantly while reading this article.
- Please acknowledge kindly your work mates, funding organizations and all helpers as well. 

**References**

- Very good referencing and good number of references. 

Is the rationale for developing the new software tool clearly explained?

Yes

Is the description of the software tool technically sound?

Yes

Are sufficient details of the code, methods and analysis (if applicable) provided to allow replication of the software development and its use by others?

Yes

Is sufficient information provided to allow interpretation of the expected output datasets and any results generated using the tool?

Yes

Reviewer Expertise:

Energy technology

I confirm that I have read this submission and believe that I have an appropriate level of expertise to confirm that it is of an acceptable scientific standard, however I have significant reservations, as outlined above.

# Response

**Published:** 2025-08-09

**Authors:** Konstantinos Koasidis

**Affiliations:** School of Electrical and Computer Engineering, National Technical University of Athens, Zografou, Attica, Greece

**R4.1:** General comments It is a good article, and it is written well. A lot of pages for an article, but it is well if it is accepted by the Journal. If the minor comments are addressed for this article, I recommended it will be accepted based on editor’s decision. **Response: **The authors would like to thank the reviewer for their positive general assessment of the manuscript.

**R4.2:** The article language check-ups and its editing

 The language is ok, well written and not seen that much language mistakes. **Response: **We thank the reviewer for the positive comment and thorough check on the language and clarity of the manuscript.

**R4.3:** Title The title is great and it’s on the number of counts is ok, which is 15-word count. The maximum number of words shall be less than 12 and more than 6. Otherwise, the title represents the article text well even if it is a bit long one. It reflects the content of the article and its strength. **Response: **Indeed, the title is towards the higher-end of the word-count, but we deemed it necessary to clearly reflect the content of the article. Based on the journal’s “authors’ guidelines”, we confirm that the length of the title does not violate explicit guidelines.

**R4.4:** Abstract The abstract is good and it looks good, well organized and written with intellect. The abstract on well written and well reflecting the chance they have to show their passion in research of this kind. Please highlight the aims, novelties, contributions and significance of the study. Avoid using references in the abstract section. **Response: **We confirm that the abstract does not include any references. On the structure, given that this is a “software tool article”, we adapted the standard

 “Background, Methods, Results and Conclusions” format used in research articles and used the following logical flow: “Context, Gap, Added Value and novelty of the software, Methodological underpinnings, Pilot use cases to validate the tool, Concluding remarks”. Through this equivalent structure, we deem we highlight the aspects indicated in the reviewer’s comment.

**R4.5:** Keywords The 7 keywords, which are the number of counts of key words that are in the requirement limit. **Response: **We confirm that the number of keywords used (7) adheres to journal standards (8).

**R4.6: **The introduction The introduction is short, it ok, well written and good level compressive that will help reader understand the concept. It has a very good acceptability by the readers and help to understand the context of the article well. It is very ok, but the method used and the compressive paper is good. Research question should be included. A good level of references was used. The number of references can help understand well the climate change issue along with energy security. **Response: **We thank the reviewer for the feedback. A formal research question that is common in research articles might not be as relevant in a software tool article. We confirm that the research motivation (i.e., the gaps in MCDA and GDM tools) and objectives (i.e., developing a tool with added value) are clearly presented in the introduction. We revised the following sentence to explicitly make the research objective clear to the reader: *“To address this gap, the research objective of this paper is to introduce APOLLO-Live”*

**R4.7:** Methods The methods written in was long and good one, well done. The method used was good, well-organised and impressive well to highlight the necessity of this method to work on this paper. In addition, the number of references and method, which is efficient are able to give a soon compressive review paper. **Response: **We thank the reviewer for the positive evaluation of the methodological section. In principle, we agree that it is on the higher end of word count, but we believe this is justified to ensure that the methodological, technical and functional elements are adequately described.

**R4.8:** Conclusions The paper is good and it summarizes all the necessary section the article. The conclusion is very good, it is well written text, and good conclusions mentioned. It is matured and well written text that helps the reader understand the whole text is perfect. It has constructive conclusion that very goodly highlighted and showed the paper. Future direction section is quite well written. **Response: **We thank the reviewer for the positive assessment of the conclusion and future directions sections.

**R4.9:** Abbreviations and Acknowledgments Please include acknowledgment and abbreviation full list on your paper. If it is included, helps significantly while reading this article. Please acknowledge kindly your work mates, funding organizations and all helpers as well. **Response: **We confirm that the article features an acknowledgment section with explicit mentions on support received in our research, a section on funding, while a CRediT author statement is also included to ensure that the co-authors’ contributions are clearly defined. Finally, it is our understanding that the journal does not have a dedicated section in the manuscript for an abbreviation table. Nonetheless, we thoroughly checked the manuscript to ensure that all abbreviations are defined upon first use, which is a common practice, remedying instances of an abbreviation not defined.

**R4.10:** References Very good referencing and good number of references. **Response: **Thank you for the positive remark on references. They have been slightly expanded to include further webtools in the literature to better position our tool in the field. **Final Remark:** Once again, the authors would like to thank the reviewer for the thorough checks and suggestions, which helped us improve our manuscript.

# Reviewer report: Reviewer response for version 2

**DOI:** 10.21956/openreseurope.22598.r56695

**Published:** 2025-07-21

**Authors:** Tuhibur Rahman

**Affiliations:** 1 Information Technology, Deakin University, Melbourne, Victoria, Australia

The manuscript presents **APOLLO-Live**, a web-based stakeholder engagement tool for real-time, group-based decision-making in climate and energy policy deliberations. Built upon the 2-tuple TOPSIS methodology within an MCDA framework, the tool aims to support consensus-building in workshops and participatory settings. The paper is well-written, addresses a timely need, and contributes to the field of participatory decision support.

 However, several key areas require strengthening before the work is suitable for indexing.

 1.While the paper describes a useful implementation of MCDA in a web environment, its **methodological contribution is unclear**. The reliance on existing techniques (e.g., 2-tuple TOPSIS, linguistic scales) is valid, but the paper should explicitly articulate **what is new**—in either process, interface design, or application strategy. How is APOLLO-Live meaningfully different from other tools in the MCDA landscape?

 2. Both use cases (a classroom workshop and an online form with youth) offer illustrative value but are **too limited in scope and sample size** to substantiate broader claims. Please Consider adding **larger, more diverse samples** in future iterations, reporting **user experience metrics**, usability feedback, or post-workshop surveys and clearly stating that these are **pilot examples**, not generalizable findings.

 3. The authors briefly discuss strategic voting and consensus dynamics but should provide **stronger evidence or safeguards** against manipulation. Explain how the tool tracks erratic voting patterns? Discuss the **limitations of consensus metrics** when used in small or ideologically diverse groups. Clarify how the tool supports disagreement or preserves dissenting voices, especially when consensus is not the goal?

 4. The manuscript would benefit from a **comparative review** of existing GDM and MCDA tools used in similar domains (e.g., DEMO, DEXi, Web-HIPRE). A table or short section comparing APOLLO-Live’s capabilities, features, and domain applicability with similar tools would help readers understand its **relative advantages and trade-offs**.

Is the rationale for developing the new software tool clearly explained?

Partly

Is the description of the software tool technically sound?

Partly

Are sufficient details of the code, methods and analysis (if applicable) provided to allow replication of the software development and its use by others?

Partly

Is sufficient information provided to allow interpretation of the expected output datasets and any results generated using the tool?

Yes

Reviewer Expertise:

Information Technology, AI, Reinforcement Learning, Energy Transition and Climate Change, Electric Vehicles

I confirm that I have read this submission and believe that I have an appropriate level of expertise to confirm that it is of an acceptable scientific standard, however I have significant reservations, as outlined above.

# Response

**Published:** 2025-08-09

**Authors:** Konstantinos Koasidis

**Affiliations:** School of Electrical and Computer Engineering, National Technical University of Athens, Zografou, Attica, Greece

**R3.1:** The manuscript presents APOLLO-Live, a web-based stakeholder engagement tool for real-time, group-based decision-making in climate and energy policy deliberations. Built upon the 2-tuple TOPSIS methodology within an MCDA framework, the tool aims to support consensus-building in workshops and participatory settings. The paper is well-written, addresses a timely need, and contributes to the field of participatory decision support. However, several key areas require strengthening before the work is suitable for indexing. **Response: **The authors would like to sincerely thank the reviewer for their positive remarks and thorough suggestions for improvement. ****We believe that the revisions performed based on the reviewer’s report have significantly improved the quality of the manuscript.

**R3.2:** While the paper describes a useful implementation of MCDA in a web environment, its methodological contribution is unclear. The reliance on existing techniques (e.g., 2-tuple TOPSIS, linguistic scales) is valid, but the paper should explicitly articulate what is new—in either process, interface design, or application strategy. How is APOLLO-Live meaningfully different from other tools in the MCDA landscape? **Response: **The reviewer is correct that aspects of the methodological foundations of the APOLLO-Live tool are based on existing methodologies (including as correctly pointed out the 2-tuple TOPSIS, the linguistic scales, and the basis for the consensus metric used), which were implemented in the original APOLLO tool. The added value (and different elements) of this tool is twofold. First, given that the original APOLLO was asynchronous, the whole APOLLO-Live tool, including the interface and the functionalities, is markedly different in structure, usefulness and openness. Additionally, based on the reviewer’s suggestion in comment R3.5 we also provide elaborations of how the tool is different compared to other MCDA tools, notably on its built-in capabilities to facilitate synchronous GDM. Both these elements are reflected in the following addition in the manuscript: *“As such, although the methodological foundations rely on existing methods with targeted expansions, the tool itself, including the interface and its functionalities, constitute a novel contribution to the field of participatory decision support. Other commonly known MCDA webtools include DEXi, which uses hierarchical trees and qualitative rule tables, and Hiview3 and Web-HIPRE which employ value trees and functions grounded in the multi-attribute value theory. In these tools, a stakeholder can define the rule tables (in the case of DEXi) or provide quantitative weights (for Web-HIPRE this can be achieved through the Analytic Hierarchy Process). However, none of these tools offer built-in functionalities for real-time or formal preference aggregation internally. Consequently, to be useful in the context of GDM, the individual models of the stakeholders should be post-processed externally, or they should jointly build one representative model. The built-in voting, aggregation, consensus metrics and multi-round process of APOLLO-Live aim to address this gap.” * Second, a core novelty of the tool lies in a newly established process for providing feedback and tips to support consensus improvement, not part of the original which focused only on consensus measuring. We discuss how literature has implemented CRP processes so far, the bottlenecks stemming from necessary methodological choices, and the newly implemented solution. This is reflected in the following passage: *“In the original APOLLO version, no such processes were implemented, since the tool only focused on consensus measuring. Usually, in the first approach, which uses aggregations based on weighted averages, the group decision matrix and the matrices of the stakeholders are of equal dimensions and format, making them easy to compare and provide the required tips. This is not the case when first calculating the individual solution for each stakeholder and then the global group solution, due to the difference in the dimensions of the initial matrix that the stakeholders vote on and the matrices emerging along the way. For this reason, most applications of CRP opt for the former approach. Given our methodological reliance on the latter process though, as explained above, and to address this gap, in APOLLO-Live we apply a consensus support process based on tips using the following protocol, which constitutes an expansion on the existing methods used in the original APOLLO tool.”*

**R3.3:** Both use cases (a classroom workshop and an online form with youth) offer illustrative value but are too limited in scope and sample size to substantiate broader claims. Please Consider adding larger, more diverse samples in future iterations, reporting user experience metrics, usability feedback, or post-workshop surveys and clearly stating that these are pilot examples, not generalizable findings. **Response: **We agree with the reviewer that the use cases presented serve primarily to highlight the functionalities of the tool, the provision of results, and ways to interpret them. In the main text we include numerous disclaimers explicitly mentioning that the results should be treated as numerical examples and not be interpreted as generalisable findings. To further enhance this, as the reviewer indicated, we added the term *“pilot”* to accompany the previous term “use cases” in numerous instances throughout the text, crucially including in the abstract and the respective subsections, which are now termed as *“Pilot use cases”*. The remark to include post-workshop surveys and feedback on the tool, notably based on larger samples, is a valuable suggestion. So far, the non-structured feedback we received on the tool focused on beta-tests to incorporate comments and reach a stable version. However, having reached that stage, in the future research section, we added the prospect of rigorously gathering feedback from a large sample through post-workshop surveys: *“…future work on the tool includes its use in full-scale experiments with a large number of participants, including post-workshop feedback surveys to validate user experience…”*

**R3.4:** The authors briefly discuss strategic voting and consensus dynamics but should provide stronger evidence or safeguards against manipulation. Explain how the tool tracks erratic voting patterns? Discuss the limitations of consensus metrics when used in small or ideologically diverse groups. Clarify how the tool supports disagreement or preserves dissenting voices, especially when consensus is not the goal? **Response: **Thank you for these remarks and suggestions. As elaborated in the main text, we argue that the potential for some form of strategic behaviour is always part of any system that tries to aggregate individual preferences into a collective result (i.e., also drawing a conceptual parallel to Arrow’s impossibility theorem). In this case, penalties may be incorporated to discourage strategic voting. But such approaches might not yield the expected results, since such patterns are usually hard to detect and they run the risk of penalising truthful votes. As the reviewer correctly points out, this is a limitation of consensus metrics in general and especially in ideologically diverse groups, and penalties could potentially crowd out honest disagreements. For this reason, we opted not to implement penalties, which implies that the tool preserves all votes (even when disagreements exist). We elaborate on this in the revised paragraph on group behaviours. *“However, as previously discussed, convergence to a consensual result is neither guaranteed nor the only end goal. During the discussions, the emergence of disagreements is also possible, especially in cases of small or ideologically diverse groups. In this case, it is the responsibility of the organiser and moderator of the exercise to ensure that the discussions avoid escalation and conflict, but rather remain fruitful, and that the results can meaningfully inform decision makers, especially on aspects that were found difficult to bridge, or were not bridged in the end. As such consensus metrics may still be useful to reflect such differences but are limited in terms of reaching a widely accepted solution. The tool opts not to penalise dissenting voices* *at the individual level, hence preserving all viewpoints expressed.”* With this in mind, we opted for an indirect approach towards discouraging strategic voting and encouraging truthful expression via methodological choices made. We argue that it would be rather difficult to influence the results through such a strategy within the employed MCDA approach, as the input required is more complex than simple selection, and hence strategic distortion is diluted. As a safeguard, the tool includes a figure on the individual consensus of stakeholders (still anonymously), which provides a simple qualitative security over truly erratic voting patterns. Although not an explicit mathematical tracking, the number of each stakeholder presented in the figure remains the same throughout voting rounds, ensuring that continuous erratic behaviours are easier to be identified. In these cases, the awareness of the participants and the moderator can arguably be more important than any mathematical algorithm. We enhanced the following section to reflect on these: *“The methods employed within the tool encourage truthful expression and discourage strategic voting. Crucially, it would be rather difficult to influence the results through strategic behaviour within the employed MCDA approach, as the input required is more complex than simple selection, and hence strategic distortion is diluted (i.e., participants evaluate all alternatives against a set of criteria rather than selecting a preferred option). The tool also presents a figure on the individual anonymised consensus level of stakeholders (see the Operation section), which provides a simple qualitative security over truly erratic voting patterns. The anonymised identifier of each stakeholder remains the same in each voting round within this figure, to ensure an indirect tracking of continuous distortions in the form of soft social enforcement. In these cases, the awareness of the participants and the moderator can arguably be more important than any mathematical algorithm to protect the process.”*

**R3.5:** The manuscript would benefit from a comparative review of existing GDM and MCDA tools used in similar domains (e.g., DEMO, DEXi, Web-HIPRE). A table or short section comparing APOLLO-Live’s capabilities, features, and domain applicability with similar tools would help readers understand its relative advantages and trade-offs. **Response: **As hinted in our response to comment R2.2, we added a short paragraph comparing APOLLO-Live to existing MCDA webtools in the literature, namely, DEXi, Hiview3, Web-HIPRE. Given the different scopes of these tools (i.e., not designed for synchronous GDM), we opted for a paragraph instead of a table as it was deemed more appropriate to highlight the differences. The following section has been added: *“Other commonly known MCDA webtools include DEXi 62, which uses hierarchical trees and qualitative rule tables, and Hiview3 63 and Web-HIPRE 64 which employ value trees and functions grounded in the multi-attribute value theory. In these tools, a stakeholder can define the rule tables (in the case of DEXi) or provide quantitative weights (for Web-HIPRE this can be achieved through the Analytic Hierarchy Process). However, none of these tools offer built-in functionalities for real-time or formal preference aggregation internally. Consequently, to be useful in the context of GDM, the individual models of the stakeholders should be post-processed externally, or they should jointly build one representative model. The built-in voting, aggregation, consensus metrics and multi-round process of APOLLO-Live aim to address this gap.”*

**Final Remark:** The authors would once more like to genuinely thank the reviewer for their helpful and constructive comments.

# Reviewer report: Reviewer response for version 1

**DOI:** 10.21956/openreseurope.21217.r52787

**Published:** 2025-04-11

**Authors:** Phoebe Koundouri

**Affiliations:** 1 Technical University of Denmark, Lyngby, Denmark

**revise and resubmit**

 The authors present a model that can aid real time and asynchronous decision making, presenting two case studies. The tool presents an interesting interface that can be both useful for the organizers and the participants. However, my main concern has to do with the group dynamics that can be triggered by the model.

 1) The authors have created a model for “consensus reaching” (among others). However, it is not clear how the methodology presented promotes consensus. The following bullets illustrate the same major issue, presented in three different ways.

- The authors claim that: “…in our case, convergence lies entirely in the participants’ behaviour, which is not always cooperative as implied in all simulated cases, where some form of compromise is always assumed”. Indeed, convergence cannot be guaranteed, but it needs to be promoted through the model structure. The model indeed offers useful group-focused results and can be used to feed an interesting discussion between rounds, but how does this lead to consensus? A fruitful discussion helps the stakeholders improve their understanding of their own and the others’ preferences, but this can also lead to a disagreement.
- Specifically, there are two main questions: why would a voter change their vote and why would that change be towards the most popular opinion in the group (mainstream group opinion)?
- Suppose a voter A, a vector of 5 options *{* *x* *1* *,* *x* *2* *,…,* *x* *5* *}*  and a vector of preferences *{* *u* *A* *x* *1* *w* *,* *u* *A* *x* *2* *w* *,…,* *u* *A* *x* *5* *w* *}* , representing the utilities that voter A obtains from each of the options, when this option wins (noted as “w”) after a number of rounds. Finally suppose that a voter A presents the following preferences: *u* *A* *x* *1* *<* *u* *A* *x* *2* *<* *u* *A* *x* *3* *<* *u* *A* *x* *4*  and *u* *A* *x* *4* *>* *u* *A* *x* *5* . Namely, if there was one round and A was honest, A would vote *x* *4* , targeting on *u* *A* *x* *4* . However, as there is no penalty inserted in the model presented, nor any other mechanism that could lead to consensus, the model can lead to anti-coordinating behavior, which is interestingly the opposite of the goal of the paper. Actually, in the first rounds voter A has strong motivation to vote *x* *5*  (even though they would prefer *x* *4* ). This way the results of the “model” will present biased results, making the players with other preferences (such as *x* *1* *,* *x* *2* *,* *x* *3* *)*  move towards *x* *4* *,* *x* *5*  in order to reach consensus. This process, known as strategic voting, is actually encouraged by the model. Anonymity also makes it easier for such players to determine the outcome of the discussions. 

2) In page 17, trying to obtain generalizable outcomes about youth opinions in climate change from 5 participants should be strongly avoided. At the same time, we should have in mind that these 5 students are not randomly chosen. They are the students that “with no academic incentives provided” have been agreed to participate. Does environmental awareness make them participate? Is it their good relationships with the model developers that makes them participate? There are so many parameters that affect youth behavior.

 3) The structural characteristics of the model that make it particularly useful for energy and climate policy discussions are not clear through the manuscript.

Is the rationale for developing the new software tool clearly explained?

No

Is the description of the software tool technically sound?

Yes

Are sufficient details of the code, methods and analysis (if applicable) provided to allow replication of the software development and its use by others?

Yes

Is sufficient information provided to allow interpretation of the expected output datasets and any results generated using the tool?

Yes

Reviewer Expertise:

Environmental & Natural Resource EconomicsSustainable Development Water & Marine Resources Energy & Green Transition .Climate Economics – Climate risk, carbon pricing, policy impact.Behavioral & Experimental Economics Interdisciplinary Systems Research

I confirm that I have read this submission and believe that I have an appropriate level of expertise to confirm that it is of an acceptable scientific standard, however I have significant reservations, as outlined above.

# Response

**Published:** 2025-07-04

**Authors:** Konstantinos Koasidis

**Affiliations:** School of Electrical and Computer Engineering, National Technical University of Athens, Zografou, Attica, Greece

**Comment R2.1.** The authors present a model that can aid real time and asynchronous decision making, presenting two case studies. The tool presents an interesting interface that can be both useful for the organizers and the participants. However, my main concern has to do with the group dynamics that can be triggered by the model.

**Response**:  The authors would like to sincerely thank the reviewer for their constructive and thorough remarks. We are confident that the revisions performed in line with these comments significantly improved the quality of the manuscript, notably regarding elaborations on consensus and potential group dynamics arising. In this response, we annotate with italics exact sections from the manuscript that have been added or updated.

**Comment R2.2. **1) The authors have created a model for “consensus reaching” (among others). However, it is not clear how the methodology presented promotes consensus. The following bullets illustrate the same major issue, presented in three different ways.

**Response: ** The reviewer raises some very interesting remarks in this set of comments. In the principle that comments R2.2-R2.5 are positioned within a broader argument, but also constitute dedicated comments, we will respond to them individually but also following a consistent narrative as well (i.e., two dedicated paragraphs on group dynamics have been inserted in the theoretical underpinnings section of the manuscript to discuss these elements; see more details in the responses below). On a high-level note, as the reviewer acknowledges, consensus is not the single goal of the tool—with a central goal being the prioritisation of alternatives. However, even within the notion of consensus, reaching one is always preferable/ideal but not a goal in itself. The tool provides tips and suggestions to potentially help improve consensus, but whether these are taken on board lie entirely in the participants’ hands. Even in the cases though that consensus is not reached, we deem these results interesting to understand why consensus was not reached, how the opinions of groups of participants differ, and consequently inform decision making. This is why we opted for the Krohling and Campanharo solution process for group decision making, which calculates individual solutions on top of the aggregated solution. Based on this discussion, we added the following disclaimer: *“We note, however, that consensus reaching is not necessarily the ultimate objective. As such, even in the cases that the process ends without consensus, the results can still be of added value to inform decision- or policy-making on different viewpoints in the group that are difficult to bridge. Following the solution process introduced by Krohling and Campanharo, the aim was to ensure that the individual and grouped solutions are also calculated to accompany the results produced by the tool.”* Additionally, to better reflect on this, we have kept the term “consensus reaching” in broader discussions as it is the standard term used in this field, but we replaced it with terms such as “ *consensus-based decision aid*” and “ *Consensus support*” (in text and in the title of manuscript as well) when referring to the purpose of the tool itself.

**Comment R2.3: **The authors claim that: “…in our case, convergence lies entirely in the participants’ behaviour, which is not always cooperative as implied in all simulated cases, where some form of compromise is always assumed”. Indeed, convergence cannot be guaranteed, but it needs to be promoted through the model structure. The model indeed offers useful group-focused results and can be used to feed an interesting discussion between rounds, but how does this lead to consensus? A fruitful discussion helps the stakeholders improve their understanding of their own and the others’ preferences, but this can also lead to a disagreement.

**Response:** We agree with the reviewer that the deliberations and discussions taking place between rounds of voting could indeed help stakeholders improve their understanding of their own and the others’ preferences, adding that they could also discover lines of thinking not initially considered, which could lead them to change their voting. We made the following addition to the theoritical underpinnings section (see also response to R2.4): *“These theoretical underpinnings bring forward group dynamics that should be considered. A core issue lies in understanding why the opinions of participants could change by participating in such activities. The tool offers group-focused suggestions that can be used to feed discussions between rounds, which can help stakeholders improve their understanding of their own and the others’ preferences and beliefs, as well as be exposed to different viewpoints not previously thought of or largely considered. These mechanisms could influence participants towards changing their opinions and consequently their votes. Crucially, the ability of deliberations to change opinions has long been established in the literature (see for example Fishkin’s work on “Deliberative polling”, or Chong & Druckman’s work on framing and deliberation). Our first use case presents an example of how such behaviours could manifest in the tool and alter the results, changing the order of the alternatives, rather that force the first-round opinion of the majority.”* However, it is also true that deliberations could lead to disagreements, which is an inherent part of any deliberation. For this reason, we explicitly mentioned in the previous response (R2.2) that reaching consensus is not the only goal. It is the responsibility of the organiser and moderator of the exercise to ensure that the discussion remains fruitful even within disagreements, and that the results can meaningfully inform decision makers. Based on this, we added the following: *“However, as previously discussed, convergence to a consensual result is neither guaranteed nor the only end goal. During the discussions, the emergence of disagreements is also possible. In this case, it is the responsibility of the organiser and moderator of the exercise to ensure that the discussions avoid escalation and conflict, but rather remain fruitful, and that the results can meaningfully inform decision makers, especially on aspects that were found difficult to bridge, or were not bridged in the end.”*

**Comment R2.4**: Specifically, there are two main questions: why would a voter change their vote and why would that change be towards the most popular opinion in the group (mainstream group opinion)?

**Response:** Following on our response to comment R2.3, we believe that deliberations and discussions in between voting, help participants reflect on their beliefs, the ones of others, as well as potentially be made aware of ideas and considerations not previously thought of. Through these mechanisms, it is quite possible that participants could alter or change their votes. Many foundational papers discuss the possibility of participants’ opinions changing, from Fishkin’s work on “Deliberative Polling” showing that in structured deliberations opinions often change (see for example Fishkin and Luskin, 2005), to Chong and Druckman (2007) arguing that, through a properly developed framing, it was possible to move public opinion. In our first numerical example, we also present a case that this happened with participants getting introduced to ideas not previously considered, hence altering the ranking. On the latter point, it is not necessary that opinions need to change towards the most popular opinions. It is also possible that the result (i.e., ranking) in the second round of voting is different to that in the first one. The first numerical example serves as a case for that, with the first two options altering places in the ranking.  Based on these, and your helpful remark, we made the following additions: *“These theoretical underpinnings bring forward group dynamics that should be considered. A core issue lies in understanding why the opinions of participants could change by participating in such activities. The tool offers group-focused suggestions that can be used to feed discussions between rounds, which can help stakeholders improve their understanding of their own and the others’ preferences and beliefs, as well as be exposed to different viewpoints not previously thought of or largely considered. These mechanisms could influence participants towards changing their opinions and consequently their votes. Crucially, the ability of deliberations to change opinions has long been established in the literature (see for example Fishkin’s work on “Deliberative polling”, or Chong & Druckman’s work on framing and deliberation). Our first use case presents an example of how such behaviours could manifest in the tool and alter the results, changing the order of the alternatives, rather that force the first-round opinion of the majority.”*

- Fishkin, J. S., & Luskin, R. C. (2005). Experimenting with a democratic ideal: Deliberative polling and public opinion. Acta politica, 40, 284-298.
- Chong, D., & Druckman, J. N. (2007). Framing public opinion in competitive democracies. American political science review, 101(4), 637-655.

**Comment R2.5**: Suppose a voter A, a vector of 5 options  and a vector of preferences , representing the utilities that voter A obtains from each of the options, when this option wins (noted as “w”) after a number of rounds. Finally suppose that a voter A presents the following preferences:  and . Namely, if there was one round and A was honest, A would vote  , targeting on . However, as there is no penalty inserted in the model presented, nor any other mechanism that could lead to consensus, the model can lead to anti-coordinating behavior, which is interestingly the opposite of the goal of the paper. Actually, in the first rounds voter A has strong motivation to vote  (even though they would prefer ). This way the results of the “model” will present biased results, making the players with other preferences (such as  ) move towards  in order to reach consensus. This process, known as strategic voting, is actually encouraged by the model. Anonymity also makes it easier for such players to determine the outcome of the discussions. 

**Response:** This is an interesting thought experiment that helps us clarify aspects of the methodological underpinnings of the tool, as well as advance the discussion on strategic or even manipulative voting. First, the approach followed is based on the TOPSIS method, which provides an order of preference, meaning that the end result is a ranking of alternatives, rather than a “winner-takes-all” approach. Although, it is observed that in a ranking-based result people tend to focus on the first place, this is inherently wrong, as useful information is deduced also from the distance between alternatives, as well as from those alternatives placed in the bottom rank. Second, TOPSIS is a multi-criteria decision aid method, meaning that the participants evaluate the impact of all alternatives on a series of criteria, rather than select one preferred alternative. This makes “rigging the system” more difficult. Following the nomenclature of the reviewer’s comment, this strategic participant would have to provide alternative 5 with very high evaluations across all criteria and alternative 4 with very low. But this would cause their individual consensus to be very low and, if this erratic voting behaviour was expressed in the deliberations, it would be fairly easy for the group to isolate said participant (the individual consensus is shown to participants in Figure 4b; the figure is anonymous but there is space to identify persistent erratic voting behaviours. On the other hand, the participant could also evaluate alternative 4 relatively high, but then this would have a much smaller impact on the result diluting the distortion. Also, this voting pattern is an admittedly distorted but rather realistic expression of their preference. Third, the thought experiment assumes that voters have a fixed utility for a given result (e.g., dominance of a solution, which given the MCDA setup, is not usually the case). However, as discussed in points R2.3 and R2.4, opinions and hence preferences and utilities could change as part of the process.  Overall, the potential for some form of strategic behaviour is always part of any system that tries to aggregate individual preferences into a collective result. Hence, we view this remark over the tradeoff between truthful expression and strategic influence as not a weakness of the APOLLO-Live tool, but a general criticism of collective decision-making. However, drawing a conceptual parallel with Arrow’s impossibility theorem, we argue that rarely can any mathematical tool be foolproof against all types of potential manipulation.  We also argue that our tool encourages truthful expression and discourages strategic voting. As previously explained, it would be rather difficult to influence the results through such a strategy within the employed MCDA approach, as the input required is more complex than simple selection, and hence strategic distortion is diluted. What is more, the tool includes a figure on the individual consensus of stakeholders (still anonymously), which provides a simple qualitative security over truly erratic voting patterns. In these cases, the awareness of the participants and the moderator can arguably be more important than any mathematical algorithm.  We elaborate on this very interesting discussion in the final paragraph of the theoretical underpinnings section in Methods: *“Finally, the potential for some form of strategic behaviour from participants, who may try to take advantage of the system to promote self-interests, is always part of any system that tries to aggregate individual preferences into a collective result. Drawing a conceptual parallel to Arrow’s impossibility theorem, we argue that rarely can any mathematical tool be foolproof against all types of potential manipulation. The tradeoff between truthful expression and strategic influence is not only a weakness of the APOLLO-Live tool, but of collective decision-making in general. The methods employed within the tool encourage truthful expression and discourage strategic voting. Crucially, it would be rather difficult to influence the results through strategic behaviour within the employed MCDA approach, as the input required is more complex than simple selection, and hence strategic distortion is diluted (i.e., participants evaluate all alternatives against a set of criteria rather than selecting a preferred option). The tool also presents a figure on the individual anonymised consensus level of stakeholders (see the Operation section), which provides a simple qualitative security over truly erratic voting patterns. In these cases, the awareness of the participants and the moderator can arguably be more important than any mathematical algorithm to protect the process.”* Finally, a potential suggestion to include penalties for uncoordinated behaviours as an explicit method to discourage strategic voting might not yield the expected results. Such patterns are usually hard to detect (e.g., even in the thought experiment how do we differentiate a participant that prefers alternative 5 over someone who promotes alternative 5 only to converge to alternative 4). As such penalties come at a disadvantage of their own, as they run the risk of penalising truthful votes. There is a very recent paper that aimed to address manipulative and manipulated behaviours in GDM (Zhong et al., 2025), but the authors acknowledge that simple weight penalties are insufficient and require more complex methods to address, while such complex methods still rely on participants’ willingness to make subjective adjustments, and the potential of a cluster of manipulators influencing results remains as a gap.  Based on upcoming advancements in this field, future versions of the tool could attempt to incorporate such mechanisms for addressing manipulative behaviour. We added this in the future research part of the conclusions: *“…as well as incorporation of different solving techniques (e.g., MCDA methods other than the 2-tuple Group TOPSIS used in GDM), and consensus reaching mechanisms, as well as explicit methods for incorporating manipulative behaviours”*

- Zhong, X., Liu, F., Du, Z., & Wan, Q. (2025). A Two-Stage Consensus Model Incorporating Manipulative and Manipulated Behaviors for Large Group Decision-Making under Social Network Environment. Applied Soft Computing, 113240.

**Comment R2.6:** 2) In page 17, trying to obtain generalizable outcomes about youth opinions in climate change from 5 participants should be strongly avoided. At the same time, we should have in mind that these 5 students are not randomly chosen. They are the students that “with no academic incentives provided” have been agreed to participate. Does environmental awareness make them participate? Is it their good relationships with the model developers that makes them participate? There are so many parameters that affect youth behavior.

**Response:** The reviewer is correct that the discussions presented on the use cases should not be misinterpreted as generalisable findings. The use cases serve primarily as numerical examples, highlighting the functionalities of the tool. In the first version, we had already provided some disclaimers on this. Acknowledging that these required strengthening, in this version we further expanded on these to ensure transparent reflections on the commentary provided.  In particular, we first expanded on the existing disclaimer in the intro of the “Use Cases” section, right before the start of the two use cases, further stating that the commentary provided serves as an example of how results of the tool from potential representative exercises can be translated to concrete decision-aid findings: *“As such, any commentary on the results is primarily intended to showcase how results of the tool from future exercises can be interpreted and translated into relevant findings—if representativeness is achieved—rather than generalisable findings on their own” * Crucially, the whole paragraph serving as an example of the interpretation of results from APOLLO-Live in the first use case mentioned by the Reviewer was heavily edited. Notably, we added a disclaimer to ensure that the caveat of a small sample is clear, we ensured clarity that the commentary serves as an example of how one could potentially engage with the tools’ results by toning down statements, and we also offered examples to analysts of how to interpret results in general, rather than focus on the results themselves: *“The following reflections are not generalisable due to the limited sample but can illustrate how APOLLO-Live can be used analytically in future exercises. In our case, they also highlight that there may be value in discussing alternatives ranked in the last places, since such results are usually left with limited commentary in ranking-based exercises. In particular, participation in climate activism actions ranked in the bottom, despite youth movements gaining traction as a means of protest ^46^, with evidence showing such initiatives are overall viewed positively by the youth ^47^. Using results from the APOLLO-Live engagement process and cross-validating them with literature can open up several interpretive avenues—further supported by participant discussions—that demonstrate the kind of reflective engagement the tool can support. In this indicative case, even among the young generation, there may be largely different opinions on the effectiveness of a certain action; this may reflect the opinion of only a small sample, but it may also underscore the need to significantly expand deliberation and co-creation activities to include the youth and capture broader viewpoints of the engaged groups. Also, the actions provided to the participants as options are not mutually exclusive, meaning that results merely reflect prioritisation rather than selection. This is usually the case for most MCDA questionnaires based on ranking; hence analysts should be careful in their interpretations. Considering the small sample caveat, these elaborations act as an example of how outcomes of the tool should be dynamically interpreted through active discussions with the participants, instead of strictly based on the quantitative results.”*

**Comment R2.7: **3) The structural characteristics of the model that make it particularly useful for energy and climate policy discussions are not clear through the manuscript

**Response: ** As we acknowledge in the conclusions, when developing the tool, our guiding principles focused on how it could be applied to problems related to energy and climate policy. However, the tool can be meaningfully applied in other fields. Still, as the Reviewer correctly points out, the structural choices making the tool explicitly tailored to energy and climate policy decisions could be improved. As such, throughout the theoretical underpinnings section of the manuscript, we added specific requirements/attributes incorporated that we enhance the tool’s link to energy and climate policy.  Notably on the choice of MCDA as the core part of the solution algorithm we added: *“Relying on MCDA, the tool aligns with the complexity of climate and energy trade-offs, since decisions are often based on environmental, economic, social, and technical criteria.”* On the selection of TOPSIS and linguistic values, we now mention: *“Through these methodological choices, the tool enables comparisons and ranking under value-based judgments, which are essential for navigating energy policy objectives (e.g., which investments should be implemented or prioritised according to stakeholders and what is their potential impact).”* On the use of 2-tuples, we explain that: *“This semi-quantitative approach allows the integration of both qualitative and quantitative criteria, reflecting the reality of energy and climate policy decisions where not all criteria are strictly numerical.”* And on the choice of approach to enable the calculation and comparison of individual, grouped and collective solutions, we mention that: *“This design enables integrating uncertainty and stakeholder diversity considerations in decision and policy support, which is common in climate-related topics.”* We acknowledge that these four requirements/attributes and the associated structural choices (i.e., criteria trade-offs, comparisons and ranking under value-based judgments, qualitative and quantitative criteria, uncertainty and stakeholder diversity) are not unique in energy and climate policy decisions independently. but collectively, they are requiring characteristics to be able to capture the energy and climate policy space. We now explicitly make this argument in the conclusions: *“Additionally, when designing and developing the tool, our guiding principles focused on how it could be applied to problems related to energy and climate policy. Consequently, structural choices of the model aimed to capture relevant requirements and attributes such as criteria trade-offs, comparisons and ranking under value-based judgments, qualitative and quantitative dimensions, uncertainty and stakeholder diversity. Although these requirements are not unique in energy and climate policy decisions independently, collectively integrating such aspects is necessary to be able to capture problems in the energy and climate policy space.  However, this does not exclude potential uses of the tool across completely different domains, again with the caveat that following the standard MCDA format in designing future use cases is necessary.”*

**Final remark: **Once again, the authors would like to thank the reviewer for deeply engaging with our research, and we hope the changes made, especially on methodological clarifications related to consensus and group dynamics, address their concerns.

# Reviewer report: Reviewer response for version 1

**DOI:** 10.21956/openreseurope.21217.r52784

**Published:** 2025-04-04

**Authors:** George Tsihrintzis

**Affiliations:** 1 University of Piraeus, Piraeus, Greece

The authors present APOLLO-Live, a webtool for engaging stakeholders in online discussions. The authors initially focus their efforts on the use of the tool on climate change and energy policy-related group decision making; however, the same tool can be used in more general settings and for engaging stakeholders in other disciplines too. Two use cases are also included in the authors' paper.

 Overall, the development of APOLLO-Live is expected to fill in a gap in the group decision making tools, as current tools are oriented more towards asynchronous rather than synchronous (live) interactions.

 The authors also identify some limitations of the current version of APOLLO-Live, which they can improve in future versions of it. In addition to the limitations identified by the authors, it is a good idea for them to also look at issues of explainability of the suggestions made by the tool, as explained suggestions may be easier to adopt by interested parties.

Is the rationale for developing the new software tool clearly explained?

Yes

Is the description of the software tool technically sound?

Yes

Are sufficient details of the code, methods and analysis (if applicable) provided to allow replication of the software development and its use by others?

Yes

Is sufficient information provided to allow interpretation of the expected output datasets and any results generated using the tool?

Yes

Reviewer Expertise:

Artificial intelligence, machine learning, decision support systems, recommender systems, artificial intelligence-empowered software, artificial intelligence-empowered information systems

I confirm that I have read this submission and believe that I have an appropriate level of expertise to confirm that it is of an acceptable scientific standard.

# Response

**Published:** 2025-07-04

**Authors:** Konstantinos Koasidis

**Affiliations:** School of Electrical and Computer Engineering, National Technical University of Athens, Zografou, Attica, Greece

**Comment R1.1.** The authors present APOLLO-Live, a webtool for engaging stakeholders in online discussions. The authors initially focus their efforts on the use of the tool on climate change and energy policy-related group decision making; however, the same tool can be used in more general settings and for engaging stakeholders in other disciplines too. Two use cases are also included in the authors' paper. Overall, the development of APOLLO-Live is expected to fill in a gap in the group decision making tools, as current tools are oriented more towards asynchronous rather than synchronous (live) interactions.

**Response: **The authors would like to thank the reviewer for their positive recommendation and remarks. 

**Comment R1.2. **The authors also identify some limitations of the current version of APOLLO-Live, which they can improve in future versions of it. In addition to the limitations identified by the authors, it is a good idea for them to also look at issues of explainability of the suggestions made by the tool, as explained suggestions may be easier to adopt by interested parties.

**Response: ** This is a very interesting suggestion. The current version of the tool already includes some explanations, but these mostly orient towards elaborating on the metrics used, as well as providing simple descriptions of the figures. Dynamic explanations and additional suggestions, potentially leveraging artificial intelligence and acknowledging caveats associated with such solutions, could indeed provide added value for future versions. As suggested, we reflect on these in the final sections of the conclusions, with the following addition: *“Finally, although efforts have been made to provide users of the tool with elaborations on the results, these are mostly static and focus on the definitions of the metrics used and the figures presented. In future versions, the dynamic explainability of the model’s results can be considered, also leveraging breakthroughs in explainable artificial intelligence”*
